# Bidirectional histone-gene promoters in *Aspergillus*: characterization and application for multi-gene expression

**DOI:** 10.1186/s40694-019-0088-3

**Published:** 2019-12-09

**Authors:** Jakob K. H. Rendsvig, Christopher T. Workman, Jakob B. Hoof

**Affiliations:** Department of Biotechnology and Biomedicine, Technical University of Denmark, 2800 Kongens Lyngby, Denmark

**Keywords:** Aspergillus, Bidirectional promoters, Histone, Motifs, Heterologous expression, Biosynthetic gene clusters, Malformin, Non-ribosomal peptide synthetase, Adenylation domain

## Abstract

**Background:**

Filamentous fungi are important producers of enzymes and bioactive secondary metabolites and are exploited for industrial purposes. Expression and characterization of biosynthetic pathways requires stable expression of multiple genes in the production host. Fungal promoters are indispensable for the accomplishment of this task, and libraries of promoters that show functionality across diverse fungal species facilitate synthetic biology approaches, pathway expression, and cell-factory construction.

**Results:**

In this study, we characterized the intergenic region between the genes encoding histones H4.1 and H3, from five phylogenetically diverse species of *Aspergillus*, as bidirectional promoters (P*h4h3*). By expression of the genes encoding fluorescent proteins mRFP1 and mCitrine, we show at the translational and transcriptional level that this region from diverse species is applicable as strong and constitutive bidirectional promoters in *Aspergillus nidulans*. Bioinformatic analysis showed that the divergent gene orientation of *h4.1* and *h3* appears maintained among fungi, and that the P*h4h3* display conserved DNA motifs among the investigated 85 Aspergilli. Two of the heterologous P*h4h3*s were utilized for single-locus expression of four genes from the putative malformin producing pathway from *Aspergillus brasiliensis* in *A. nidulans*. Strikingly, heterologous expression of *mlfA* encoding the non-ribosomal peptide synthetase is sufficient for biosynthesis of malformins in *A. nidulans*, which indicates an iterative use of one adenylation domain in the enzyme. However, this resulted in highly stressed colonies, which was reverted to a healthy phenotype by co-expressing the residual four genes from the putative biosynthetic gene cluster.

**Conclusions:**

Our study has documented that P*h4h3* is a strong constitutive bidirectional promoter and a valuable new addition to the genetic toolbox of at least the genus *Aspergillus*.

## Background

Certain species of filamentous fungi particularly of genus *Aspergillus* are widely applied for production of primary metabolites (organic acids), industrial enzymes, pharmaceutical proteins, and bioactive secondary metabolites (SMs). Regardless of the product type and the origin, the availability of suitable fungal promoters is an important consideration for controlling gene expression in new hosts. For this reason, characterization of constitutive or inducible promoters that function in a heterologous context increases the application range of filamentous fungi as expression hosts. Researchers in the last few decades have identified a small number of promoters that have become de facto standards for expression in *Aspergillus*, most notably the constitutive P*gpdA* [1] and P*tef1* [2], the inducible P*alcA* [3] and P*glaA* [4], and the synthetic Tet-on expression system [5]. While sufficient for simple gene-expression tasks, this promoter library may become limiting when applied to biosynthetic pathways with multiple genes. Moreover, heterologous promoters that provide a similar transcriptional strength to endogenous promoters are attractive, as they reduce the risk of integrating the expression cassettes in the locus of an endogenously derived promoter.

Heterologous expression of SM pathways is important for elucidation of biosynthetic pathways originating from non-tractable hosts or for successful activation of a silent pathway [6]. Hence, it requires the expression of all pathway-contributing genes, which are often organized as a physically coherent biosynthetic gene cluster (BGC). The expression of the full BGC in the native or heterologous host may be accomplished by overexpressing a gene-cluster specific transcriptional activator associated with the BGC [7–9]. However, a significant number of BGCs do not contain a gene encoding a transcription factor (TF) and only ~ 30% of the gene clusters display co-expression with contiguous TFs [10]. In addition, the overexpression of activating TFs may have the drawback of an indirect activation of other BGCs compromising the analysis of the specific gene cluster under investigation. An alternative approach to ensure activation of the BGC is by the functional expression of each gene. This requires multiple promoters reacting to the same stimuli, since consecutive use of the same promoter would be prone to genetic instability and genome defense mechanisms like repeat-induced point mutations [11].

Therefore, we set out to exploit the reservoir of stable bidirectional promoters, which would ease the heterologous expression of BGCs. One example is the histones H4.1 and H3 encoded by the genes *h4.1* and *h3*. These genes are divergently transcribed and the intergenic region therefore likely serves as a bidirectional promoter (P*h4h3*). Moreover, in filamentous fungi the orientation of these genes appear conserved and are most often present as single copies in the genome, separated by 799 bp in the case of *Aspergillus nidulans*. The P*h4h3* of *A. nidulans* (*h4.1*; *hhfA* and *h3*; *hhtA*) was first characterized by Ehinger et al. [12], who showed that the expression from each of the respective ends were approximately equal and regulated in accordance with the cell cycle. This was also observed for both copies of *h4.1* (*HHF1*, *HHF2*) and *h3* (*HHT1*, *HHT2*) in the yeast *Saccharomyces cerevisiae* [13, 14]. The endogenous promoters of histone encoding genes have previously been used for various purposes in *Penicillium funiculosum* [15], *Aspergillus oryzae* [16], and the yeast *Arxula adeninivorans* [17]. Recently, the P*h4h3* from the yeast *Pichia pastoris* was used to generate a library of synthetic bidirectional promoters [18]. The use of other natural bidirectional promoters in filamentous fungi has so far been ascribed to the inducible promoters controlling expression of *pcbAB*/*pcbC* from *Penicillium chrysogenum* [19], *niaD*/*niiA* and *stcA*/*stcB* from *A. nidulans* [20].

Owing to the great importance of H4.1 and H3 proteins in chromatin structure and the apparent widespread conservancy in the divergent orientation, we hypothesized that P*h4h3* from different species would be functional across species within a fungal genus. Moreover, we aimed at investigating if the employment of multiple P*h4h3*s of different species could be applied for one-locus expression of heterologous BGCs. To accomplish this, we expressed the two genes encoding fluorescent proteins mRFP1 and mCitrine in *A. nidulans* using five different P*h4h3*s; the endogenous *A. nidulans* P*h4h3*, and the heterologous P*h4h3*s from *Aspergillus niger*, *Aspergillus flavus*, *Aspergillus clavatus*, and *Aspergillus terreus*. These five species represent five different sections of genus *Aspergillus*; Nidulantes, Nigri, Flavi, Clavati, and Terrei, and therefore represent a phylogenetic set of heterologous promoters applied for expression analysis. The relative expression strength of both ends of each promoter was qualitatively accessed by fluorescence microscopy and quantitatively analyzed by RT-qPCR during solid-state and submerged cultivations at different growth phases. Lastly, the P*h4h3* of *A. niger* and *A. clavatus* were applied for heterologous expression of the putative BGC responsible for biosynthesis of the malformin compound family from *A. brasiliensis* in *A. nidulans* [21].

## Results

### Sequence analysis of *Aspergillus* P*h4h3* promoters

The DNA sequences from orthologous intergenic regions of *h4.1* and *h3* (P*h4h3*) were collected from 85 genome sequenced *Aspergillus* species and five fungi representing other important fungal genera; *Neurospora crassa*, *Trichoderma reesei*, *Penicillium rubens*, *Agaricus bisporus*, and *S. cerevisiae* for sequence comparison. The *h4.1* and *h3* genes of each species were inferred from protein sequence homology to *hhfA* (*h4.1*, in this study *h4* for simplicity) and *hhtA* (*h3*) of *A. nidulans* using BLASTp [22]. The extracted P*h4h3* sequences and gene prediction details are listed in Additional file 1: Table S1, and further described in Additional file 2.

In brief, the 85 curated *Aspergillus* P*h4h3* sequences were analyzed to generate a multiple sequence alignment with a 790 bp consensus sequence. The alignment revealed several highly conserved DNA motifs, located predominantly in the region − 100 to − 200 nt relative to the translational start site (ATG) at each end of the bidirectional promoters (Additional file 2: Figure S2). The consensus sequence was scanned by 177 fungal TF binding motif models from JASPAR [23], resulting in 81 matches (52 different TFs) with a score above 9 (Additional file 3: Tables S3–S4).

**Fig. 1:**
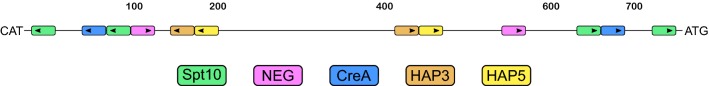
Putative regulatory motifs in P*h4h3*. The 790 bp consensus P*h4h3* with the putative motif positioning shown and motif 5′–3′ directionality indicated by an arrow. The promoter length and the translational start site (ATG/CAT) at each promoter end are shown. The distribution of motifs is to scale, while motif lengths are not. The motif sequence found in the consensus P*h4h3* are listed in Additional file 3, and the sequence conservation of each motif is shown in the Logo-plot in Additional file 2: Figure S2. Note; the two CreA sites positioned downstream of the Spt10 site have four and three overlapping nucleotides, respectively. The two pairs of HAP3/HAP5 sites (15 nt per site) have 10 overlapping nucleotides, incl. the core CCAAT motif. The two outer most Spt10 motifs are 51 and 58 nt upstream of the respective promoter end

In the search for regulatory motifs involved in the bidirectional expression, we primarily focused on binding sites with two or more putative matches in reverse orientation located in opposite ends of the P*h4h3* consensus sequence. In addition, we included regulatory motifs described to be involved in histone gene expressions in humans and *S. cerevisiae* [24–26]. Firstly, expression of core histone-encoding genes in *S. cerevisiae* occurs primarily during S-phase activated through the DNA binding of Spt10 and subsequent recruitment of S-phase-specific Spt21 [27, 28]. Analysis of the consensus P*h4h3* sequence for presence of the yeast Spt10 motif [29] (retrieved from YeTFaSCo; motif #1322) revealed four putative Spt10 binding sites ordered pairwise (separated by 63 and 59 nt) in opposite orientation in each end of the promoter (Fig. 1), as also reported for *S. cerevisiae* [25]. Orthologues of yeast Spt10 were identified by protein sequence homology in each of the 85 Aspergilli (< 2E−53) (data not shown). Secondly, the negative cell cycle regulation of the gene expression partly derives from the NEG region and associated motif [26, 30]. Analysis of the consensus P*h4h3* sequence with the NEG motif [14] revealed three low-scoring matches (Fig. 1), positioned similarly as in *S. cerevisiae* [30]. Thirdly, the NF-Y complex regulates core histone gene expression in humans via the motif 5′-CCAAT-3′ [24], and interestingly we found two pairs of HAP3/HAP5 motifs (homologs of human NFY B and C) in the consensus P*h4h3* oriented towards the promoter ends (Fig. 1). Additionally, two putative binding sites for yeast MIG1-3 (fungal CreA) [31] were found located in opposite ends and in reverse orientations in the consensus P*h4h3* (Fig. 1). Lastly, the analysis also revealed several lower scoring motifs of the major G1/S cell-cycle-dependent transcription factor MBF (MluI cell cycle box binding factor) [32], which is involved in the positive regulation of histone gene transcription during the G1/S transition of the mitotic cell cycle in *S. cerevisiae* [33] (Additional file 3). In addition to the identified putative motifs of known TFs, the alignment of P*h4h3* revealed several highly conserved motifs that could not be associated with a given TF in our analysis, among others; 5′-TACAAATA-3′ resembling a TATA box, and 5′-CTCGCTTA-3′ (Additional file 2: Figure S2).

The DNA motifs discovered in P*h4h3* of Aspergilli could partially be found in the P*h4h3* of the four selected reference filamentous fungi *N. crassa*, *T. reesei*, *P. rubens*, and *A. bisporus* (Additional file 3). Collectively, we found DNA motifs in the P*h4h3* of Aspergilli from both higher eukaryotes and yeasts, potentiating shared regulatory features. Interestingly, overrepresentations of putative TF binding sites are located in the promoter region on the coding strand upstream of each histone gene (Fig. 1). Here we have presented an overview of conserved regulatory DNA motifs in the P*h4h3* of *Aspergillus*, and the dataset of P*h4h3* sequences provided in this study may spur more investigations.

### Design of strains expressing reporter genes using the bidirectional promoters

The P*h4h3* of five Aspergilli; *A. nidulans* (NID), *A. niger* (NIG), *A flavus* (FLA), *A. clavatus* (CLA), and *A. terreus* (TER), were chosen as the proof of concept promoters employed to express genes encoding the fluorescent proteins mRFP and mCitrine, with transcriptional termination by T*tef1* and T*trpC*, respectively. The promoters were defined as the intergenic regions between the genes for H4.1 and H3, respectively; *A. nidulans* (AN0734.2 and AN0733.2), *A. niger* (ANI_1_974074 and ANI_1_976074), *A. clavatus* (ACLA_021660 and ACLA_021650), *A. flavus* (AFLA_017620 and AFLA_017610), and *A. terreus* (ATEG_00587 and ATEG_00586). The P*h4h3*s had approximately the same lengths in the five species; *A. nidulans* (799 bp), *A. niger* (828 bp), *A. flavus* (821 bp), *A. clavatus* (853 bp), and *A. terreus* (822 bp).

**Fig. 2:**
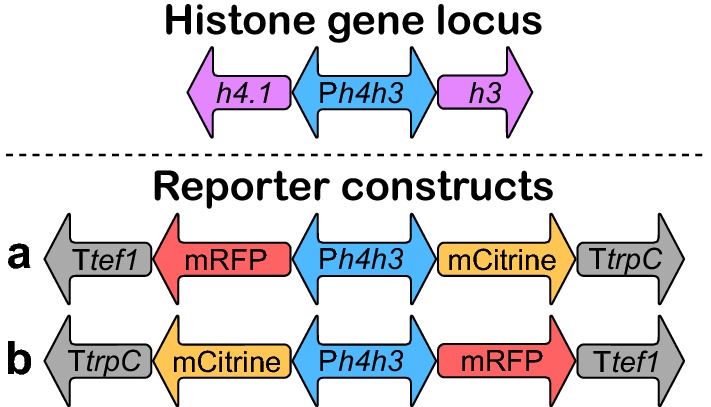
Gene orientation in the reporter expression constructs. The native histone gene locus, and the reporter constructs using the five P*h4h3*s to express mRFP and mCitrine from promoter ends P*h4*′ (left) and P*h3*′ (right) in the type *a* and type *b* orientation, respectively

Using the five P*h4h3*s, 10 constructs were made to confer expression of *mRFP*-T*tef1* and *mCitrine*-T*trpC* from either end of each P*h4h3* (Fig. 2). For comparison, two reference constructs were tested using the promoters of *gpdA* and *tef1* expressing *mRFP* with transcriptional termination by T*trpC*. The P*gpdA* and P*tef1* from *A. nidulans* had similar lengths to P*h4h3* of 836 bp and 886 bp, respectively.

Each of the 10 P*h4h3* expression constructs (Fig. 2) and the control constructs with *mRFP* controlled by P*gpdA* and P*tef1* were assembled by USER (*uracil specific excision reagent*) cloning. USER cloning is dependent on the inclusion of uracil-linked 5′ extensions of the primers used for fragment amplification, enabling the generation of complimentary overhangs and seamless assembly of DNA fragments. Verified vectors were linearized prior to transformation and gene targeting was performed in a non-homologous end joining deficient *A. nidulans* host strain targeting the integration site 4 (IS4) [34]. Biological triplicates from each transformation were validated rigorously by diagnostic PCR for correct targeted integration and to consist of homokaryotic nuclei in respect to IS4 (Additional file 4: Figure S3). Potential additional integration events of reporter constructs at the endogenous loci of P*h4h3*, T*trpC*, and T*tef1* were excluded by diagnostic PCR (Additional file 4). None of the constructed P*h4h3* reporter strains showed any morphological change compared to the reference strain (Additional file 4: Figure S4a), and we continued with evaluating the promoter performance.

### Evaluation of promoter strength by qualitative fluorescence microscopy

**Fig. 3:**
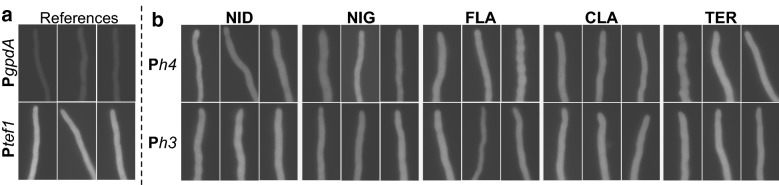
Fluorescence microscopy of strains expressing *mRFP* as reporter. Single leading hyphae from biological triplicates of **a** control promoter strains with P*gpdA* and P*tef1*, or **b** the ten P*h4h3* based strains, expressing *mRFP* under the control of either the P*h4*′ (type *a* constructs) or P*h3*′ (type *b* constructs) of the five *Ph4h3* promoters from *A. nidulans* (NID), *A. niger* (NIG), *A. flavus* (FLA), *A. clavatus* (CLA), and *A. terreus* (TER)

The 10 promoter constructs using the promoter end P*h4*′ and P*h3*′ to drive expression of *mRFP* were compared to the controls, P*gpdA* and P*tef1*, for relative promoter strength by fluorescence microscopy. Each of the strain types were cultivated in triplicate on agar-covered microscopy slides for 20 h, and exposed single representative leading hyphae are shown in Fig. 3.

Based on the observations presented in Fig. 3, all ten promoter combinations were able to drive *mRFP* expression as the mRFP production appeared close to equal in strength for all individual types of constructs as well as for the triplicates. Compared to the control-promoter constructs, the mRFP production appeared much stronger in the P*h4h3*-based expression strains than in P*gpdA*, but not as strong as the *Ptef1* strains.

An equivalent signal of mCitrine fluorescence was observed from both ends of the five P*h4h3* promoters (data not shown). However, the mCitrine signal was slightly weaker than the mRFP signal in all cases (despite expressed from the same promoter end) and was therefore not used in the promoter comparison. This confirmed that both ends of the bidirectional promoter were active during growth.

### Quantitative determination of promoter strength during solid-state cultivation

**Fig. 4:**
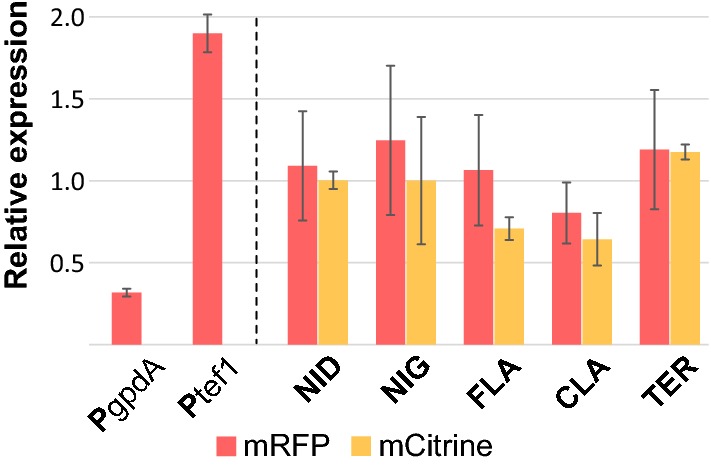
Relative expression from P*h4h3* during solid state cultivation. Relative expression by RT-qPCR on *mRFP* (red) and *mCitrine* (orange) of each of the five P*h3h4*s (NID, NIG, FLA, CLA, TER), and the references promoters, P*gpdA* and P*tef1*, see text for details

The qualitative analysis of promoter strength by protein fluorescence indicated that the bidirectional P*h4h3* promoters were of similar strength in both directions, but we also wanted to quantitatively measure the promoter strength at the mRNA level using RT-qPCR. Biomass was harvested during growth on plates (72 h) from seven strains; five strains based on the P*h4h3* promoter constructs in the type *a* orientation expressing both *mRFP* and *mCitrine* (Fig. 2) and two strains using the reference promoters, P*gpdA* and P*tef1*, to drive *mRFP* expression (see Fig. 4).

Firstly, no significant differences were observed between the relative expression levels obtained from the five P*h4h3*s of *A. nidulans* (NID), *A. niger* (NIG), *A flavus* (FLA), *A. clavatus* (CLA), and *A. terreus* (TER), when compared to the endogenous *A. nidulans* P*h4h3*. This observation was also true for the different orientations of *mRFP* and *mCitrine*, with the exception of the relative expression level of *mCitrine* (P*h3*′) from the *A. terreus* P*h3*′, which was significantly higher than *mCitrine* expressed from both the P*h3*′ of *A. clavatus* (*P *= 0.033) and *A. flavus* (*P *= 0.0048). Secondly, P*gpdA* and P*tef1* indicated a lower and higher expression level of *mRFP*, respectively, compared to the P*h4h3*s, however only the *mCitrine* level expressed from P*h3*′ of *A. clavatus* was significantly different from P*tef1* (*P *= 0.0075).

P*gpdA* is routinely used for various types of expression constructs in *A. nidulans*, and is historically approximately 2.3 kbps long [35]. In this study, we have shortened P*gpdA* to 836 bps for it to be of comparable size to the other promoters used. Therefore, the influence that this reduction in length may have for the promoter strength was examined by comparing the 836 bps version to the 2.3 kbps long P*gpdA* (P*gpdA*_2.3_), both fused to *mRFP*-T*trpC* (Additional file 4: Figure S5). RT-qPCR did not reveal a significant difference between the two promoter variants.

As described in the bioinformatics analysis, long regions of the P*h4h3* were highly conserved in Aspergilli, notably in the region 100–200 bps upstream of each end. To investigate the importance of the less conserved middle region, two constructs were made with internal truncations of the *A. nidulans* P*h4h3*, in which the middle part of the P*h4h3* was removed, specifically the middle 400 bp or 600 bp, thereby constructing two shortened versions of P*h4h3*s composed of the outermost 200 bp or 100 bp of each end (pAC1688–1689), respectively. After verification for targeted integration in *A. nidulans* IS4 and homokaryon, the strains were analyzed by fluorescence microscopy. Here, removal of the middle 400 bp from P*h4h3* decreased expression at each end slightly, while removal of the middle 600 bp abolished expression from both ends (data not shown). This may be due to the removal of the inner most Spt10 site in each end through the 600 bp truncation (Fig. 1), as the pairwise arrangement of these motifs was deemed important for the gene activation in yeast [25].

Cultivations on solid medium showed a pattern of mixed expression due to the presence of different tissue types (e.g. hyphae, apex, conidia, phialides, metulae), and hence differentiated stages of cellular dormancy. For this reason, an analysis of *A. nidulans* P*h4h3* expressing *mRFP* and *mCitrine* was carried out in submerged cultivations. Previously, our lab has used T*trpC* and T*tef1* interchangeably for expression constructs as they have appeared equally efficient. Prior to the submerged cultivations, we examined if it would be more appropriate to employ common terminators between P*h4h3* reporters and control promoters. Interestingly, exchanging the T*trpC* for T*tef1* for the control P*gpdA*_0.8_-*mRFP* expression resulted in a significant increase in relative expression, by a factor of 1.8 (P < 0.01) (Additional file 4: Figure S5). Since most of the reference promoter constructs employed T*trpC* for *mRFP* expression, a construct of the *A. nidulans* P*h4h3* expression mRFP (P*h4*′) with the T*trpC* was applied (strains NID2376-78).

### Quantitative determination of promoter strength during submerged cultivations

**Fig. 5:**
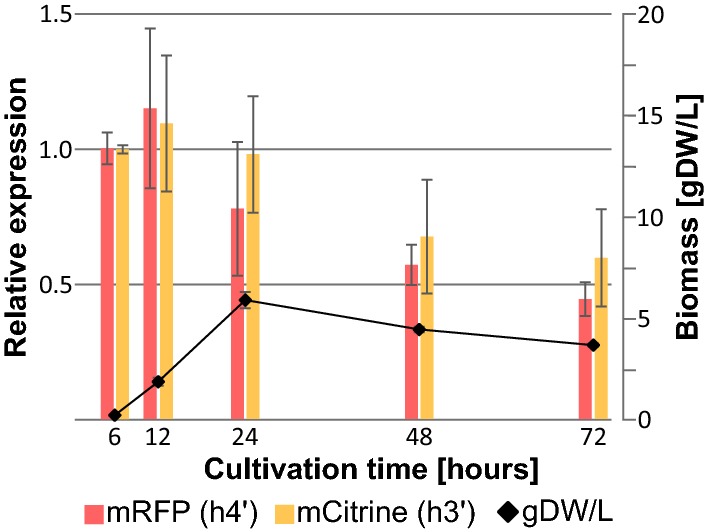
Relative expression from P*h4h3* during submerged cultivation. The relative expression level (left y-axis) from *A. nidulans* P*h4h3* expressing *mRFP* (P*h4*′, red) and *mCitrine* (P*h3*′, yellow), with comparison to the biomass concentration in gDW/L (black) (right y-axis) as a function of cultivation hours (x-axis). After 24 h, the media was depleted of glucose, followed by the onset of stationary phase

The expression profile from P*h4h3* (NID) at different growth phases during submerged cultivation was evaluated by growing the strains for up to 72 h, with sampling for RNA extraction and biomass concentration at five time-points; 6, 12, 24, 48, and 72 h. For each time-point, the relative expression levels of *mRFP* and *mCitrine* were determined by RT-qPCR, and the biomass concentration determined by dry weight (DW) measurements (Fig. 5).

Based on these results (Fig. 5), P*h4h3* was most active during the early exponential growth phase, up to 12 h, and then declined and followed the drop in biomass during onset of the stationary phase. The relative expression levels of *mRFP* (P*h4*′) and *mCitrine* (P*h3*′) at each time-point were similar, and did not display significant differences.

The reference strains expressing *mRFP* from P*gpdA* and P*tef1* were also included in this analysis, and the relative expression from these are shown in Additional file 4: Figure S6. Regardless of the promoter construct, no difference in biomass concentrations during submerged growth was observed, indicating that applying the endogenous P*h4h3* in *A. nidulans* for heterologous expression did not affect growth rate compared to using the P*gpdA* or P*tef1* (Additional file 4: Figure S7a). Normalization of the relative expression levels to the biomass concentration resulted in a similar pattern for the promoters, P*h4h3*, P*gpdA*, and P*tef1* (Additional file 4: Figure S7b–d), namely highest during early exponential phase (6 h) and declining towards the onset of stationary phase (12, 24 h), and a steady low expression level during stationary phase (48, 72 h). This may be related to the cell-cycle independent basal expression of core histones in yeast [28].

Based on the encouraging results of expression levels from P*h4h3* during both solid and submerged cultivations, we then applied multiple P*h4h3*s for single locus expression of a putative BGC.

### Heterologous expression of the malformin biosynthetic pathway

To evaluate the potential of applying P*h4h3* promoters for expression of a multi-gene pathway, the putative BGC responsible for production of malformins in *Aspergillus brasiliensis* was heterologously expressed in *A. nidulans.* Malformins are bicyclic pentapeptides with a disulfide bond produced by members of *Aspergillus* section *Nigri* and the biosynthesis of malformin in *A. brasiliensis* was recently attributed to the non-ribosomal peptide synthase (NRPS) MlfA [21]. The NRPS MlfA from *A. brasiliensis* synthesizes various malformin compounds that differ in the amino acid composition; malformin C, malformin A2, and at least one more uninvestigated malformin [21]. MlfA from *A. brasiliensis* predominantly synthesizes the compound malformin C, which has shown potential as a pharmaceutical agent by potentiating anti-cancer drugs [36], and it displays cytotoxicity in a broad range of organisms [37].

We predict that the core BGC in *A. brasiliensis* consists of the gene encoding the NRPS, *mlfA* (Aspbr1_34020), and four genes coding for three transporters (*mlfB*: Aspbr1_186232, *mlfC*: Aspbr1_134974, *mlfD*: Aspbr1_199881) and one putative thioredoxin reductase (*mlfE*: Aspbr1_161173) [21]. Note that two gene models exist for *mlfC*, therefore two parallel vectors of the residual BGC, rBGC and rBGC*, were constructed; Aspbr1_134974 (*mlfC*) and the shorter Aspbr1_334277 (mlfC*).

**Fig. 6:**
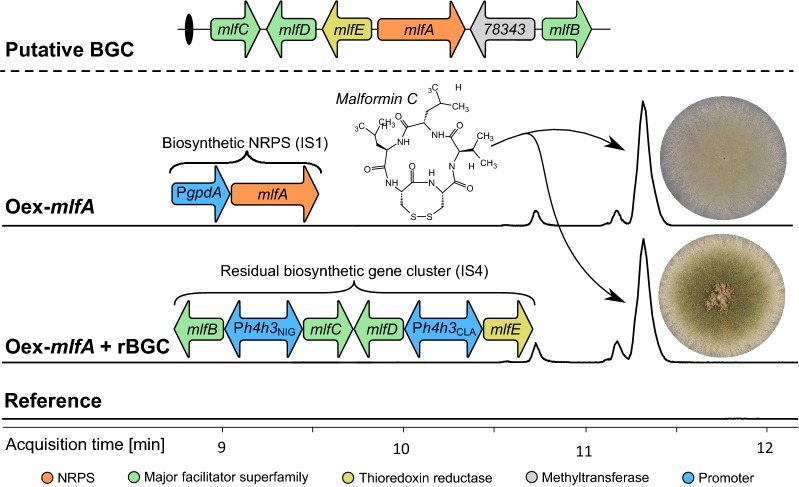
Chemical analysis of malformin producing strains. The putative malformin producing BGC is conserved between producing species in *Aspergillus* section *Nigri* and the BGC organization is shown in the top of the figure. For the chemical analysis, three EICs illustrate the malformin-production capabilities in three individual strain types. The top panel shows the expression of *mlfA* (Oex-*mlfA*) alone is sufficient to produce malformin although the production comes at a price of reduced fitness and sporulation. In the middle, the residual part of the putative BGC (rBGC or rBGC*) has been integrated, and production remains at the same level as in the top, however the phenotype has been restored to normal for both constructs. Bottom panel shows that the reference strain of *A. nidulans* do not produce malformin, as it does not possess the malformin BGC. In the bottom of the figure, the enzyme types representing the genes within the BGC are listed with color codes matching the gene arrows

The proposed BGC responsible for malformin synthesis was introduced in two steps. In the first step, the four hypothesized gene-cluster members *mlfB*-*E* were placed under control of the P*h3h4* from *A. niger* and *A. clavatus*, whereas the second step was integration of *mlfA* under control of P*gpdA*. We speculated that *mlfB*-*D* were important in malformin production as previous deletions in the native host *A. brasiliensis* resulted in extremely poor growing colonies (unpublished data), while *mlfE* could be involved in formation of the disulfide bond in malformin. The four genes were amplified as full genes extended with approximately 400 bp of their native 3′ untranslated region as terminator sequence and using the two P*h4h3* promoters they were inserted into a combined plasmid for single-locus integration in *A. nidulans* integration site 4 (IS4, Fig. 6). Integration of this residual gene cluster construct (both rBGC and rBGC*) was evaluated by diagnostic PCRs from gDNA (see Additional file 4: Figure S3). All transformants displayed the same phenotype as the reference strain, showing that introducing the two heterologous P*h4h3*s and *mlfB*-*E* did not affect growth negatively (Additional file 4: Figure S4c–d).

The chemical analysis of rBGC strains after seven days growth at 37 °C on MM revealed no changes in the chromatogram compared to reference strain, and no malformin could be detected (data not shown). To reuse the *pyrG* marker for subsequent transformation and integration of the *mlfA* expression construct, conidia were plated on MM supplemented with 5-FOA, and the resulting 5-FOA resistant colonies were analyzed for the presence of *pyrG* (Additional file 4: Figure S3d). The *pyrG* marker was reused for the secondary integration event to minimize metabolic changes in transformants that may affect the phenotypic readout.

The full-length *mlfA* was assembled from four PCR fragments by USER™ cloning as described previously [21]. However, in this case we used a vector harboring target sequences for integration in *A. nidulans* IS1 [38]. Successful integration of the P*gpdA*-*mlfA*-T*trpC* construct was evaluated by diagnostic PCRs from gDNA (Additional file 4: Figure S3). The reference host strains as well as the two strains expressing rBGC and rBGC* were transformed by linear gene-targeting constructs and three transformants were validated by diagnostic PCR. Strikingly, the strains lacking either of the *mlfB*-*E* cassettes displayed an impaired mycelial growth and sporulation phenotype showing the toxicity of malformins in *A. nidulans* (Additional file 4: Figure S4b), whereas presence of either of the two *mlfB*-*E* cassettes resulted in the healthy reference phenotype as without *mlfA* (Additional file 4: Figure S4c–d).

The verified *mlfA* overexpressing strains in the three genetic backgrounds were subjected to chemical analysis after 7 days growth at 37 °C on MM, and these data showed that all three strategies led to production of malformins. Thus, the integration of *mlfA* alone in the reference strain was sufficient to synthesize malformins in *A. nidulans*. The relative distribution in amounts of malformin C and A2 were similar to the native producer *A. brasiliensis* [21]. The presence of the putative thioredoxin reductase (*mlfE*) did not affect the relative amounts of oxidized and reduced forms of the disulfide bond in any of the detected malformin compounds.

The P*h4h3* of *A. niger* and *A. clavatus* had 59% homology with minor conserved stretches (36 nt, 25 nt, and below), limiting the risk of genetic instability and recombination between the promoters in the locus. However, we still investigated whether the locus remained intact during cultivation. Firstly, strains harboring the rBGC and rBGC* (prior to integration of *mlfA*) were propagated over a 15-day period by four times transferring spores from the peripheral zone of the colony to fresh plates, and gDNA purification from the final colonies. As expected, diagnostic-PCR analysis confirmed that the size of the expression construct was correct, with no indication of spontaneous deletions, e.g. looping out (Additional file 4: Figure S3). Secondly, strains expressing the rBGC with and without the *mlfA* in IS1 were propagated as single point inoculations in the center of a standard 9 cm petri dish with MM. Each week, the periphery of the strains had extended to the edge of the plate from where conidia were picked and transferred in the same manner to another plate. After seven successive transfers, all strains showed parental phenotypes and analysis showed that the strain still expressed the full BGC and produced malformin.

## Discussion

In this study, we aimed at demonstrating the applicability of the intergenic region between the two genes encoding histones H4.1 and H3 from multiple species of *Aspergillus* as a potential strong heterologous bidirectional promoter. Moreover, the goal was to address the relevance of such promoters for the stable and uniform expression of a BGC from a single locus. The use and characterization of promoters of histone-encoding genes have been reported previously in literature for steady and stable expression of, for example, selective markers and heterologous proteins [15–17]. Moreover, the genes *h4.1* and *h3* are highly and consistently expressed in both *A. nidulans* and *A. niger* at different pH, in both defined minimal and complex media, and during solid and submerged cultivations (personal communication Mikael R. Andersen).

We showed that the P*h4h3* from *A. nidulans*, *A. niger*, *A. flavus*, *A. clavatus*, and *A. terreus*, representing five sections of the genus *Aspergillus*, all were functional in *A. nidulans.* Based on a qualitative assessment of a biological triplicate, mRFP and mCitrine displayed a simultaneous and similar intensity under the control of all the five P*h3h4*s tested in freely extending leading-hyphae on agar microscopy slides, comparable to the levels of standard and popular fungal promoters P*gpdA* and P*tef1*. This observation was also the overall conclusion from RT-qPCR analysis of mRNA harvested from actively growing and sporulating colonies from solid media cultivations.

Among the five validated P*h4h3*s, the promoter from *A. clavatus* yielded the lowest relative expression levels in *A. nidulans*. When comparing this result to the phylogenetic analysis, i.e. the dendogram (Additional file 2: Figure S1) and the underlying distance matrix (Additional file 5: Table S2), the P*h4h3* of *A. clavatus* also display the highest distance to the P*h4h3* of *A. nidulans* (0.368). A lower distance may indicate functionality due to sequence similarity between the heterologous P*h4h3* and that of the host. However, the P*h4h3* of *A. terreus* displays the second highest difference to P*h4h3* of *A. nidulans* (0.363) and obtained among the highest expression levels. As such, the distance matrix value alone cannot predict the promoters efficiency in a heterologous *Aspergillus* host. Of the 85 P*h4h3* sequences collected in this study, 60 promoters have a distance lower or equal to that of *A. clavatus*. The experimental validation of all these promoters in other commonly applied *Aspergillus* species, and correlating the expression levels to the phylogenetic distance, may provide a significant addition of *Aspergillus* promoters.

The submerged cultivations showed that histone-expressing promoters are strongest in the exponential growth phase, and expression drops towards the onset of the stationary phase, and is minimally expressed in stationary phase. This is not surprising since cell division stops in stationary phase, inferring a lower requirement for histones. On the other hand, histone promoters are very useful for growth-coupled production either in high-density batch fermentations or, even more significantly, in continuous cultivations where a steady, high and constant expression for P*h4h3*s can be achieved. Moreover, the bidirectional P*h4h3* promoter is constitutively active during both solid state and submerged cultivation.

The use of P*h4h3* for gene expression is limited to actively proliferating cell types, since the P*h4h3* is inactive in dormant tissues, e.g. conidia. For instance, in an *A. nidulans wA*Δ *yA*Δ deletion strain displaying white conidia, we used the endogenous P*h4h3* to express the genes *wA* and *yA* from *A. nidulans*, which are responsible for yellow and green conidial pigmentation, respectively. This did not result in yellow or green conidia, but only secretion of yellow pigment (unpublished data). As such, the P*h4h3* was inactive in the dormant conidia, in agreement with the null requirement for further histone proteins, while being active in the growing fungal colony.

The divergent orientation of histone genes *h4.1* and *h3* was found beyond *Aspergillus* and is observed throughout the fungal kingdom, for example in *S. cerevisiae*, *N. crassa*, *P. rubens*, *T. reesei*, and basidiomycete *A. bisporus.* In addition to applying P*h4h3* across sections of genus *Aspergillus*, we have successfully used the P*h4h3* of *A. nidulans* for production of mRFP and mCitrine in *Penicillium brevicompactum* (unpublished), thus highlighting the expansion in potential usage of these promoters across genera, and maybe even phyla. Strikingly, the promoter length varies from 230 bp in *A. bisporus* to 2137 bp in *N. crassa* (Additional file 1: Table S1), which were the minimum and maximum lengths observed in this study. Such variation merits investigations into cross-genera functionality of P*h4h3*s and the common and genus-specific regulatory DNA motifs. Whether these P*h4h3*s of non-Aspergilli species will display sufficient transcriptional activity within their related species, or even in *Aspergillus*, remains to be seen.

The regions of low conservation in *Aspergilli* P*h4h3*, combined with the observation of a short P*h4h3* from *A. bisporus*, prompted us to truncate the P*h4h3* for several purposes. One being that smaller bio-blocks are preferred in synthetic biology. It is easier to amplify smaller fragments and amplify multiple assemblies of smaller fragments in, for example, sub-cloning steps. Another aim was that potentially an even stronger promoter could be created if the cell-cycle dependent regulation was lost. However, the results obtained in this study showed that arbitrary truncations of the central part in the intergenic region did not improve the expression of genes in either direction. The shortened promoters tested either lost some or all activity. This observation is backed by the fact that we observed highly conserved sequence regions scattered across the entire span of the promoter region. This include the Spt10 motifs of which the inner most of each pair was deleted by the 600 bp internal truncation, which led to lack of transcriptional activity, potentially due to the loss of Spt10 activation [25].

Construction of an optimized HMM model based on the P*h4h3* sequences from 85 *Aspergillus* species showed 790 conserved positions suggesting a minimal promoter could be made from the consensus 790 bps. Potentially, such a promoter would display a broad and equivalent activity level in various heterologous hosts of *Aspergillus*. The bioinformatics analysis pinpointed highly conserved regions of the P*h4h3* sequence across 85 Aspergilli, and conserved putative motifs of TFs known to regulate histone gene expression in other organisms, including Spt10, Hap3/5 and CreA. The DNA motifs discovered in P*h4h3*s of Aspergilli, could partially be found in the P*h4h3* of the other fungal species *N. crassa*, *P. rubens*, *T. reesei*, and *A. bisporus.* This could suggest that the P*h4h3*s might be able for heterologous usage in other genera with some degree of promoter activity. As previously noted, the P*h4h3* of *A. nidulans* is functional in *P. brevicompactum*, which is partly supported by the shared DNA motifs. In *S. cerevisiae*, expression of core histone encoding genes occurs primarily during S-phase through DNA binding of Spt10 and recruitment of S-phase-specific Spt21 [27] and is likewise cell-cycle dependent in *Aspergillus* [12]. The presence of putative Spt10 binding sites in P*h4h3* and orthologues of both Spt10 and Spt21 in *Aspergillus* species (data not shown), along with identification of putative Hap3/5 binding sites and NEG motifs, potentiates shared regulatory features in histone gene expression across phyla. The predicted motifs could be investigated by mutagenesis studies and pulldown-assays to examine the associated transcription factors and their role in gene regulation as activator or repressors, and as potential cell-cycle responsive elements. This might provide valuable clues into the regulation of expression of core histone genes in filamentous fungi. Collectively, if miniaturized bidirectional fungal promoters are the goal, this knowledge of functional or conserved motifs/domains might be applied to shuffle the promoter sequence elements, logically or randomly, in the aspiration of generating a synthetic promoter library, as successfully conducted for *P. pastoris* by Vogl et al. [18].

The heterologous expression of *mlfA* from *A. brasiliensis* was sufficient for production of different malformins in *A. nidulans*, with the same distribution of different malformins as the native host, albeit with a stressed and growth-impaired phenotype. This showed that malformin production is toxic to *A. nidulans*, which in itself is not surprising since malformin has been reported to be toxic to a broad range of organisms [37]. However, co-expression of the putative residual BGC, composed of three transporters and a putative thioredoxin reductase, restored the healthy parental phenotype while producing malformin. This strongly indicates that the rBGC encodes resistance factors against the toxicity of malformin, which may be supported by the fact that the organization within the BGC is conserved between the malformin-producing Aspergilli. The presence of three genes encoding transporter proteins points to a compartmentalized biosynthesis facilitating controlled production and efficient secretion. Of the four genes from the rBGC, only one of the predicted gene products, MlfB, had a homolog in *A. nidulans* (AN5329). This may explain why the *mlfA* overexpression strain could survive, while the deletion of *mlfB* in *A. brasiliensis* never resulted in viable colonies. The inclusion of the putative thioredoxin reductase did not affect the relative levels of reduced and oxidized forms of malformins during heterologous expression in *A. nidulans*, it could potentially facilitate disulfide bond formation in other target compounds in the native host. The resistance mechanisms and functions of each of the three transporters and the thioredoxin reductase warrant further investigations. Malformins are pentapeptides, yet MlfA contains four adenylation and five condensation domains [21]. The ability of *A. nidulans* to efficiently produce the same malformin types as the endogenous producer strongly indicates that MlfA is iterative in the use of one of its adenylation domains, and at that least one domain is promiscuous in the amino acid selection. With the discovery of the biosynthetic NRPS MlfA [21] and initial investigations in the BGC functions presented here, the stage is set for further studies and optimizations in malformin production, and ultimately the establishment of a fungal cell factory geared for production of this valuable compound family.

To the best of our knowledge, there has been no previous reports on the use of bidirectional heterologous P*h4h3*s for expression of BGCs. The integration of two heterologous P*h4h3*s in *A. nidulans* did not affect growth or morphology of the transformants. This indicates that sufficient amounts of regulatory proteins are present to support functional production levels of histones H4.1 and H3 from the endogenous P*h4h3*. Often BGCs contain more than five genes, and may have up to 25 genes as in sterigmatocystin/aflatoxin biosynthesis [39]. In this extreme case, a controlled expression with multiple P*h4h3*s in the genome may have a limit, where it influences growth, but this remains to be determined. To facilitate expression of larger gene clusters, one may envision combinatory usage of P*h4h3*s with the self-cleaving A2 peptide for polycistronic expression [40], which was elegantly applied for expression of four biosynthetic genes in *A. nidulans* from a single promoter [41]. The use of P*h4h3*s for uniform expression of transporters and tailoring enzymes could also be practical when the synthase or synthetase expression is regulated by a tunable promoter, so the pool of enzymes are available for production when the main scaffold is produced.

The equivalent high expression levels from the investigated P*h4h3*s may be disadvantageous for differential expression of pathway enzyme encoding genes and hereby optimizing production levels. Bidirectional promoters developed for *P. pastoris* by Vogl et al. [18] collectively displayed a 61-fold range of expression levels, thereby enabling optimization of the four pathway genes expressed. Such optimizations are also possible in filamentous fungi, e.g. using P*h4h3*s in combination with a weaker mono- or bidirectional promoters. For this approach, promoter strengths always should be benchmarked against a standard promoters as presented here and elsewhere, e.g. *pcbAB*/*pcbC* from *P. chrysogenum* [42].

Using constitutive strong heterologous monodirectional promoters in same manner as presented with the P*h4h3*s would be possible as long as the heterologous promoters have similar expression profiles in the host. For one-locus BGC expression, it will depend on the sequence homology regarding genetic stability. In future applications, the use of heterologous promoters such as P*h4h3* in a single locus will be a balance of choosing the strongest options while keeping the sequence identity of the multiple P*h4h3*s low enough, e.g. less than 90% identity, as issues of genetic instability may arise from single locus integration of multiple P*h4h3*s.

## Conclusion

In *Aspergillus*, the intergenic region of histone genes *h4.1* and *h3* has proven to be a strong constitutive bidirectional promoter (P*h4h3*) with equivalent expression levels from the two ends. Expression levels from heterologous P*h4h3*s in *A. nidulans* are comparable to that of the endogenous P*h4h3*, P*gpdA* and P*tef1* during solid-state cultivation. The P*h4h3* is active during submerged cultivation with an expression pattern similar to that of P*gpdA* and P*tef1* at the different growth stages. The P*h4h3*s from genus *Aspergillus* contain a number of conserved DNA motifs, which likely have regulatory functions. Two heterologous P*h4h3*s were applied for genetically stable single-locus integration of four genes from the malformin biosynthetic pathway of *A. brasiliensis*. The heterologous expression of *mlfA* alone was sufficient for malformin production in *A. nidulans* indicating that one of its four adenylation domains is iterative. However, expression of the entire putative BGC offers the heterologous host protection from the toxicity of malformin.

## Methods

### Strains and cultivations

The *A. nidulans* strain NID1 (*argB2*, *pyrG89*, *veA1*, *nkuA*Δ) [43], was used as host for all expression-cassette integrations. Genomic DNA (gDNA) from NID1, *A. niger* (ATCC1015), *A. clavatus* (NRRL1), *A. flavus* (NRRL 3357), and *A. terreus* (NIH2624), and *A. brasiliensis* (CBS101740) was isolated using FastDNA SPIN Kit for Soil DNA extraction kit (MP Biomedicals, USA). All strains employed and created in this study are listed in Additional file 6: Table S7. *Escherichia coli* strain DH5α was used as host for plasmid propagations.

All strains were cultivated using solid and liquid glucose based minimal medium (MM) (1% w/v) glucose, 1× nitrate salt solution [44], 0.001% (w/v) thiamine, 1× trace metal solution [45], 2% (w/v) agar for solid medium. Media was supplemented with 10 mM uridine (Uri), 10 mM uracil (Ura), and 4 mM l-arginine (Arg) when required. Solid MM + Arg + Uri + Ura plates containing 5-fluoroorotic acid (5-FOA, 1.3 mg/mL) were used in counter-selecting *pyrG*. For transformation media (TM), glucose was replaced with 1 M sucrose.

### PCR for fragment production and strain verification

PCR was used for production of fragments for USER™ cloning and strain validation, and primers are listed in Additional file 6: Table S8. Fragments for USER™ cloning were amplified using the Phusion U Hot Start PCR Master Mix (Thermo Scientific) and 0.4 µM primers (Integrated DNA Technologies, IDT), < 10 ng gDNA or 20 ng plasmid. PCR for strain verification used standard 50 µL PCR reactions including; 1 U PfuX7, 1× Phusion HF buffer (New England Biolabs, USA), 0.2 mM dNTPs, 0.4 µM primers, < 10 ng gDNA. All reactions were performed on a T100 Thermal Cycler (Bio-Rad) with initial denaturation (98 °C for 5 min) followed by 35 cycles {98 °C for 30 s; 58–62 °C for 30 s (depending on the primer pair), 72 °C 1–5 min (using 1 kb/min)} and final elongation (72 °C for 10 min).

### Vector construction

All plasmids are listed in Additional file 6: Table S9 and two vectors; pAC223 (pU2005-4) and pAC1190 (pU2005-1) served as basis for USER vector constructions [38] employing the ampicillin resistance marker and *E. coli oriC*. The fungal part of the vectors contain: selection marker *pyrG* (orotidine-5′-phosphate decarboxylase) from *A. fumigatus* under control of its native promoter and terminator flanked by a direct repeat of 282 bp [46]; a PacI/Nt.BbvCI cassette for USER cloning mediated expression cassette insertion; two 1 kb targeting sequences for integration sites 1 [38] and 4 [34] in pAC1190 and pAC223, respectively. The assembled gene-targeting substrates were flanked by SwaI sites for linearization. The *A. nidulans* IS4 is located on chromosome II in the intergenic region of AN4252 and AN4251 based of high transcriptional activity of the neighboring genes [34].

The P*h4h3* of *A. nidulans* (FGSC A4) was defined as the intergenic region between the translational start sites (ATG) of the genes encoding histone H4.1 (AN0734.2) and H3 (AN0733.2). The homologs in the other species were identified by performing a BLASTp search [22] for homologous proteins. The complete list of the histone encoding genes and the intergenic P*h4h3* sequences applied in this study is provided in Additional file 1: Table S1. Plasmids harboring gene cassettes of P*h4h3* and reference promoters expressing *mRFP1* [47] and *mCitrine* [48], and the malformin BGC were generated by amplifying PCR fragments and USER cloning according to the scheme and functionalities presented in Additional file 6: Table S10.

For the heterologous expression of the malformin BGC in *A. nidulans* it should be noted that the P*h4h3* from *A. niger* and *A. clavatus* were used to build the expression cassette for the rBGC of three putative *major facilitator superfamily* (MFS) transporters; *mlfB* (Aspbr1_186232, *mlfC* (Aspbr1_134974), and *mlfD* (Aspbr1_199881), and one putative thioredoxin reductase *mlfE* (Aspbr1_161173). All genes included their native terminators (estimated at ~ 400 bp length) and had 5′ overhangs matching the P*h4h3* of *A. niger* (*mlfB* and *mlfC*) and *A. clavatus* (*mlfD* and *mlfE*), see Fig. 6. Due to their native head-to-head orientation, *mlfC* and *mlfD* were amplified as single fragment. Note that two gene models exist for *mlfC*, therefore two parallel vectors, rBGC and rBGC*, were constructed; Aspbr1_134974 (*mlfC*) and the shorter Aspbr1_334277 (mlfC*).

The vector containing *mlfA* (Aspbr1_34020) was constructed as described in Theobold et al. [21], but specifically *mlfA* was under control of the 2.3 kb *gpdA* promoter and *trpC* terminator of *A. nidulans*, with 1 kb targeting sequences for *A. nidulans* IS1 [38].

### Promoter sequence alignment

All fungal genome sequence data retrieved from the Joint Genome Institute (genome.jgi.doe.gov) [49] and AspGD (aspgd.org) [50]. Homologous promoter sequences were identified as described in previous section for 85 Aspergillus species and 5 non-*Aspergillus* fungi (*N. crassa*, *P. rubens*, *T. reesei*, *A. bisporus* and both copies from *S.* *cerevisiae*). The DNA for these 91 P*h4h3* regions was extracted for further sequence analysis. Initial multiple sequence alignments (MSA) were performed using Clustal W (version 2.1) [51] and showed that the non-*Aspergillus* species shared only very limited sequence conservation. For this reason, only the 85 *Aspergillus* sequences were used to define the P*h4h3* alignment. An initial Clustal W alignment was used to fit a hidden Markov model (HMM) using HMMER (version 3.2.1) [52]. As the 5′ and 3′ ends of the alignment were not conserved for a number of species, but were included in the HMM, an iterative refinement was performed to identify the start and end of the conserved region. The initial HMM with 835 positions was used to re-align the 85 sequences using hmmalign and the ‘–trim’ option. This was repeated until the consensus sequence converged (after 15 iterations) resulting in an optimized 790 position HMM. This HMM was then visualized as a DNA sequence logo using Skylign [53]. The consensus sequence from the optimized HMM was extracted using ‘hmmemit -C –minl 0.25 –minu 0.5′.

### Genetic transformation

Protoplast formation and transformation was done as previously described [46, 54]. Transformation into NID1 protoplasts was conducted for all gene-targeting constructs intended for expression of fluorescent proteins, *mlfA*, or the rBGC and rBGC* constructs. Three individual and double resteaked transformants for each construct were verified by rigorous diagnostic PCR on purified gDNA, validating the targeted integration and testing for homokaryons and heterokaryons (Additional file 4: Figure S3). This was possible with primer pairs amplifying either an intact integration site or disrupted locus due to the integration of the gene-expression cassette.

The gene-targeting substrates based on rBGC and rBGC* were integrated in *A. nidulans* IS4 in NID1. Two transformants of each type were validated by PCR employing purified gDNA genotyping (Additional file 4: Figure S3a–c), and one of each was subsequently cultivated on 5-FOA for recycling of the *pyrG* marker (Additional file 4: Figure S3d). Protoplasts of these strains, named rBGC and rBGC*, respectively, were transformed with the *mlfA* expression construct and validated (Additional file 4: Figure S3). All strains expressing genes from the malformin BGC were analyzed by chemical extraction, as described previously [21].

### RNA extraction, cDNA synthesis, RT-qPCR and data analysis

RNA was extracted, using the RNeasy Plus Mini Kit (Qiagen), from biomass harvested from 3-day cultivations on solid medium and 6, 12, 24, 48 and 72 h submerged cultivations. The cDNA was synthesized using the SensiFAST cDNA Synthesis Kit (Bioline) according to manufacturer’s recommendations. RT-qPCR conducted using the SensiFAST SYBR No-ROX Kit (Bioline) according to manufacturer’s recommendations, on a CFX Connect (BioRad) using the software BioRad CFX Manager 3.1, and white 96 well RT-qPCR plates and clear lids (VWR International). RT-qPCR was conducted using primers targeting mCitrine (P71 + P72; 280 bp), mRFP (P73 + P74; 133 bp), and actin-encoding gene *actA* (AN6542) (P75 + P76; 132 bp). Active splicing the *actA* mRNA was confirmed by gel electrophoresis by determining an introns-free band of 132 bp compared to the gDNA band of 189 bp. RT-qPCR data was analyzed according to the method described by Pfaffl 2001 [55]. Reaction efficiencies were determined using cDNA from a single strain at five concentrations (0.5, 2.5, 12.5, 64, and 320 ng per 20 µL reaction) and calculating the slope between the Ct-values and the log10 of the cDNA concentration [55]. The reaction efficiencies calculated and applied were: 1.982 (*actA*), 1.999 (mRFP), and 1.948 (mCitrine). Statistical analysis was performed using a *t*-test with two-tailed homoscedastic distribution, calculated from the ΔΔCt-values. All error bars represent the standard error of the mean.

### Fluorescence microscopy

Fresh spores suspensions (10 µL of ~ 10^5^ spores/mL) were inoculated on glass slides with 0.5 mL solid MM (1% agar) + Arg and incubated for ~ 20 h in petri dishes in micro-perforated bags at 37 °C. A cover slide and immersion oil were applied and photos taken with a 100× objective and exposure times 200 ms (normal filter) or 750 ms (YFP and RFP filters), using a Nikon Eclipse E1000 fluorescence microscope equipped with a QImaging Retiga Exi camera.

## Supplementary information

**Additional file 1.** Identification of P*h4h3*. **Table S1.** Identification of histone *h4.1* and *h3* genes in 85 *Aspergillus* species and reference fungi by BLASTp of *A. nidulans* H4.1 and H3. Curated usage of gene models and prediction of the P*h4h3* sequence.

**Additional file 2.** Ph4h3 sequence analysis. **Figure S1.** Dendogram of P*h4h3* sequences from 85 Aspergilli. **Figure S2.** Logo plot of the alignment of P*h4h3* from 85 Aspergilli.

**Additional file 3.** Bioinformatic analysis of DNA motifs. **Table S3.** All results of JASPAR analysis. **Table S4.** Results of JASPAR analysis with score above 9. **Table S5.** Best hit of YeTFaSCo Spt10 in each of the 85 P*h4h3* sequences. **Table S6.** Best eight hits of YeTFaSCo Spt10 in the consensus P*h4h3* sequence. Additional motif results JASPAR analysis in reference fungi.

**Additional file 4.** Supplementing experimental data. **Figure S3.** PCR validation strategy. **Figure S4.** Pictures of fungal colonies. **Figure S5.** Relative expression from promoters during solid-state cultivation. **Figure S6.** Relative expression from reference promoters during submerged cultivation. **Figure S7.** Biomass concentration during submerged cultivation and relative expression from promoters normalized to biomass concentration.

**Additional file 5.** P*h4h3* dendogram distance matrix. **Table S2.** The distance matrix generated from the alignment of 85 Aspergilli P*h4h3*.

**Additional file 6.** Tables of Strains, Primers, Plasmids and USER fragments. **Table S7.** Strains used in this study. **Table S8.** Primers used in this study. **Table S9.** Plasmids used in this study. **Table S10.** USER fragments applied in this study.

## Data Availability

All promoter sequences are available in Additional file 1. Majority of bioinformatics results provided in Additional files 2, 3, 5. Strains, RT-qPCR data, fluorescent images, and chemical analysis data are available upon request.

## Abbreviations

P*h4h3*: promoter of *h4.1* and *h3*
mRFP1: monomeric red fluorescent protein 1
RT-qPCR: reverse transcription quantitative polymerase chain reaction
IS: integration site
SM: secondary metabolite
NRPS: non-ribosomal peptide synthetase
BGC: biosynthetic gene cluster
rBGC: residual biosynthetic gene cluster
TF: transcription factor
JGI: joint genome institute
HMM: hidden Markov model
MSA: multiple sequence alignments
DW: dry weight
USER: uracil specific excision reagent
