# Supplementary material for: Bidirectional histone-gene promoters in Aspergillus: characterization and application for multi-gene expression
Source: Fungal Biol Biotechnol. 2019 Dec 9;6:24. doi: 10.1186/s40694-019-0088-3 (PMC6900853; doi:10.1186/s40694-019-0088-3)
Supplement: Supplementary file 2 — Additional file 2. Ph4h3 sequence analysis. Figure S1. Dendogram of Ph4h3 sequences from 85 Aspergilli. Figure S2. Logo plot of the alignment of Ph4h3 from 85 Aspergilli. [file 40694_2019_88_MOESM2_ESM.docx]

**Additional file 2.** P*h4h3* sequence analysis.

**Sequence analysis of *Aspergillus* P*h4h3* promoters**

The DNA sequences from orthologous intergenic regions of *h4.1* and *h3* (P*h4h3*) were collected from 85 genome sequenced *Aspergillus* species and five fungi representing other important fungal genera; *Neurospora crassa*, *Trichoderma reesei*, *Penicillium rubens*, *Agaricus bisporus*, and *S. cerevisiae* for sequence comparison. The *h4.1* and *h3* genes of each species were inferred from protein sequence homology to *hhfA* (*h4.1,* in this study *h4* for simplicity) and *hhtA* (*h3*) of *A. nidulans* using BLASTp [1]. In some cases, the predicted *h4* or *h3* gene did not correspond to the known RNA transcript or a translation initiation site (ATG). In these cases, Fgenesh *ab initio* gene models were used to identify the gene ortholog. The extracted P*h4h3* sequences and gene prediction details are listed in Additional file 1, Table S1. Note; in seven cases, the fungal species names associated with the genome sequences received from JGI were either outdated or misspelled. The species names provided by JGI were used here for ease of study replication, however, the correct names are provided below, with the outdated species names in parenthesis; *A. cavernicola* (*A. amylovorus*), *A. jaipurensis* (*A. indicus*), *A. spinulosporus* (*A. neoechinulatus*) [2], *A. luchuensis* (*A. kawachii*) [3], *A. luteovirescens* (*A. bombycis*) [4], *A. melleus* (*A. neoauricomus*) (pers. comm. J.C. Frisvad and barcode sequencing, see Additional file 2), *Penicilliopsis zonata* (*A. zonatus*) [5], *A. stercorarius* (*A. stercoraria*). The remaining fungal species are named in accordance with newest taxonomical literature [2,6,7] and fungal databases (IndexFungorum.org, AspergillusPenicillium.org, and JGI).

The 85 curated *Aspergillus* P*h4h3* DNA sequences were analyzed to generate a multiple sequence alignment, which revealed several highly conserved motifs, located predominantly in the region -100 to -200 nt relative to the ATG start codon of each end of the bidirectional promoters (Additional file 2, Fig. S2). Overall, the alignment showed a median conservation of ~79% over the 790 alignment positions where more than 46 sequences could be aligned (>55% of sequences). The 790 bp consensus sequence from the optimized HMM model was scanned by 177 fungal TF binding motif models from JASPAR [8], resulting in 81 matches (52 different TFs) with a score above 9 (Additional file 4, Table S3-S4). In the search for regulatory motifs involved in the bidirectional expression, we primarily focused on binding sites with two or more putative matches in reverse orientation located in opposite ends of the P*h4h3* consensus sequence. In addition, we included regulatory motifs described to be involved in histone gene expressions in humans and *S. cerevisiae*, including those of the human nuclear transcription factor Y (NF-Y) complex [9], the yeast histone acetyltransferase Spt10 [10], and the NEG motif [11]. Only motifs displaying several conserved nucleotide positions in the P*h4h3* alignment Logo plot were included in the manuscript and figures.


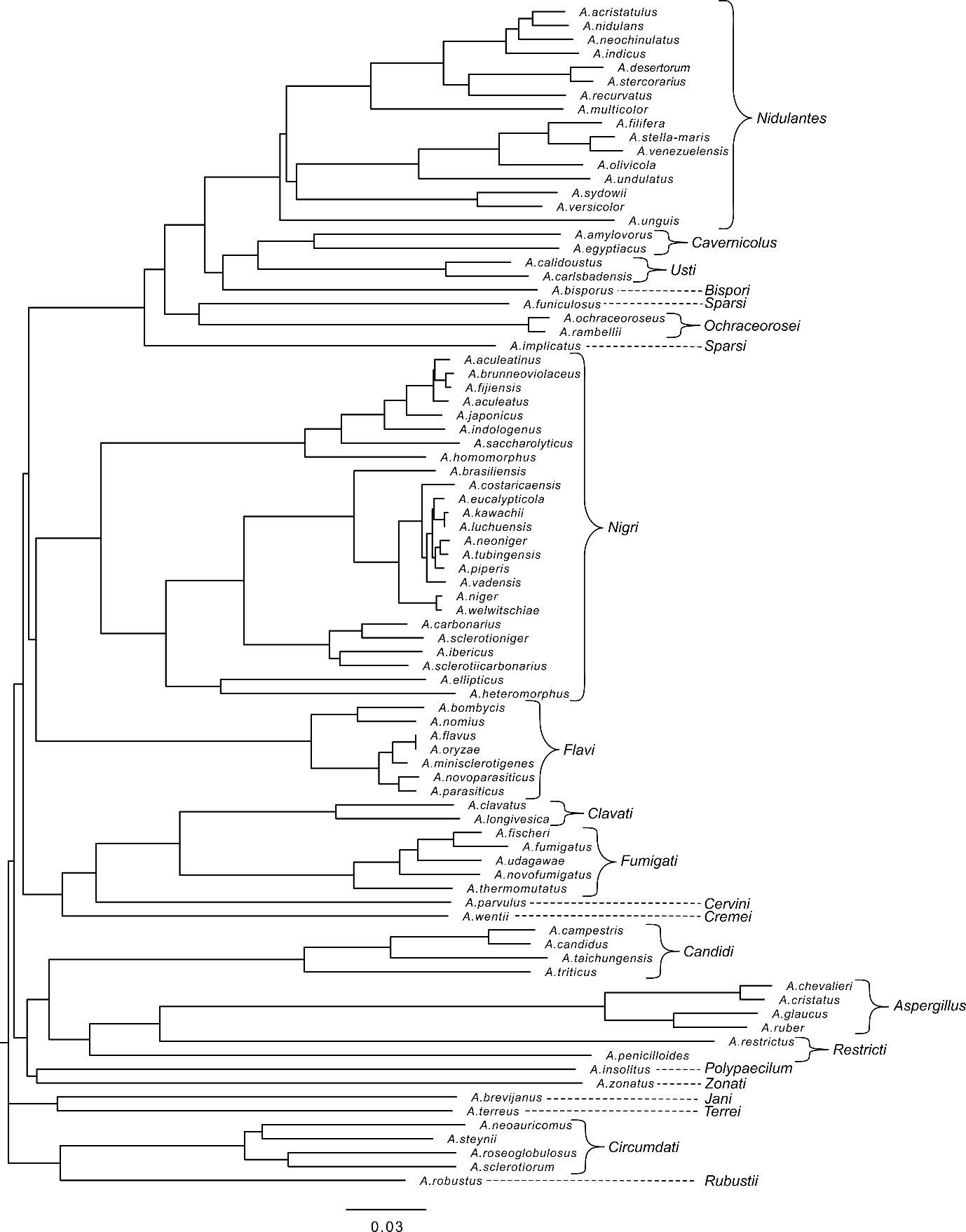


**Fig. S1. Dendogram of P*h4h3* sequences from 85 Aspergilli.** A dendogram build from the optimized alignment of the P*h4h3* sequences from 85 Aspergilli. Annotated are the individual species name (as retrieved from JGI), and the Section names; *Nidulantes*, *Cavernicolus*, *Usti*, *Bispori*, *Sparsi*, *Ochraceorosei*, *Nigri*, *Flavi*, *Clavati*, *Fumigati*, *Cervini*, *Cremei*, *Candidi*, *Aspergillus*, *Restricti*, *Polypaecilum*, *Zonati*, *Jani*, *Terrei*, *Circumdati*, and *Rubustii*. The P*h4h3* sequences of species from the same Section cluster together, as indicated with a curly bracket. For Sections in which only a single species belongs, this is indicated by a dotted line. Note; the two species of Section *Sparsi* do not cluster together, and are both indicated with dotted lines (*A. funiculosus* and *A. implicatus*).

**
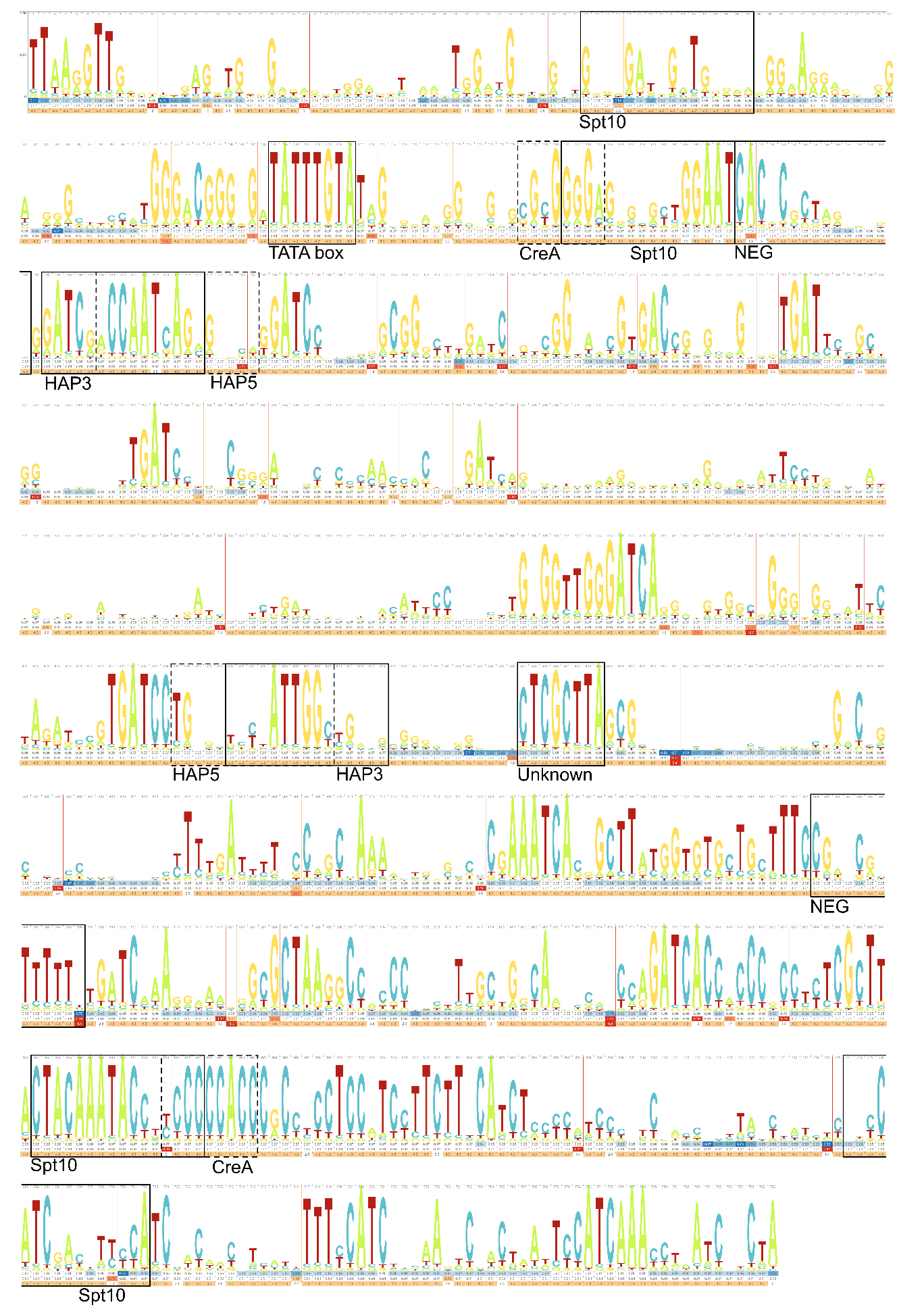
**

**Fig. S2. Logo plot of the alignment of P*h4h3* from 85 Aspergilli**. Alignment Logo plot of the (+) of the 85 P*h4h3* sequences from *Aspergillus*; therefore the first ~half of the promoter sequence is the non-coding strand. The sequence of P*h4h3* is defined as the intergenic regions between the translational start sites (ATG) of histone genes *h4.1* and *h3.* For the motifs described in main manuscript (Fig. 1), the motif sequence and its conservation is highlighted by a surrounding box with its motif name. For overlapping motifs; one box is shown in dotted lines.

**References for Additional file 2**

1. Altschup SF, Gish W, Miller W, Myers EW, Lipman DJ. Basic local alignment search tool. J Mol Biol. 1990;215:403–10.

2. Chen AJ, Frisvad JC, Sun BD, Varga J, Kocsubé S, Dijksterhuis J, et al. *Aspergillus* section *Nidulantes* (formerly *Emericella*): Polyphasic taxonomy, chemistry and biology. Stud Mycol. 2016;84:1–118.

3. Hong SB, Lee M, Kim DH, Varga J, Frisvad JC, Perrone G, et al. *Aspergillus luchuensis*, an industrially important black *Aspergillus* in east Asia. PLoS One. 2013;8:1–9.

4. Frisvad JC, Hubka V, Ezekiel CN, Hong SB, Nováková A, Chen AJ, et al. Taxonomy of *Aspergillus* section *Flavi* and their production of aflatoxins, ochratoxins and other mycotoxins. Stud Mycol. 2019;93:1–63.

5. Kocsubé S, Perrone G, Magistà D, Houbraken J, Varga J, Szigeti G, et al. *Aspergillus* is monophyletic: Evidence from multiple gene phylogenies and extrolites profiles. Stud Mycol. 2016;85:199–213.

6. Chen AJ, Hubka V, Frisvad JC, Visagie CM, Houbraken J, Meijer M, et al. Polyphasic taxonomy of *Aspergillus* section *Aspergillus* (formerly *Eurotium*), and its occurrence in indoor environments and food. Stud Mycol. 2017;88:37–135.

7. Samson RA, Visagie CM, Houbraken J, Hong S-B, Hubka V, Klaassen CHW, et al. Phylogeny, identification and nomenclature of the genus *Aspergillus*. Stud Mycol. 2014;78:141–73.

8. Khan A, Fornes O, Stigliani A, Gheorghe M, Castro-Mondragon JA, Van Der Lee R, et al. JASPAR 2018: Update of the open-access database of transcription factor binding profiles and its web framework. Nucleic Acids Res. 2018;46:D260–6.

9. Koessler H, Kahle J, Bode C, Doenecke D, Albig W. Human replication-dependent histone H3 genes are activated by a tandemly arranged pair of two CCAAT boxes. Biochem J. 2004;384:317–26.

10. Eriksson PR, Mendiratta G, McLaughlin NB, Wolfsberg TG, Marino-Ramirez L, Pompa TA, et al. Global regulation by the yeast Spt10 protein is mediated through chromatin structure and the histone upstream activating sequence elements. Mol Cell Biol. 2005;25:9127–37.

11. Osley MA, Gould J, Kim S, Kane M, Hereford L. Identification of sequences in a yeast histone promoter involved in periodic transcription. Cell. 1986;45:537–44.
