# Supplementary material for: Bidirectional histone-gene promoters in Aspergillus: characterization and application for multi-gene expression
Source: Fungal Biol Biotechnol. 2019 Dec 9;6:24. doi: 10.1186/s40694-019-0088-3 (PMC6900853; doi:10.1186/s40694-019-0088-3)
Supplement: Supplementary file 4 — Additional file 4. Supplementing experimental data. Figure S3. PCR validation strategy. Figure S4. Pictures of fungal colonies. Figure S5. Relative expression from promoters during solid-state cultivation. Figure S6. Relative expression from reference promoters during submerged cultivation. Figure S7. Biomass concentration during submerged cultivation and relative expression from promoters normalized to biomass concentration. [file 40694_2019_88_MOESM4_ESM.docx]

**Additional file 4.** Supplementing experimental data.


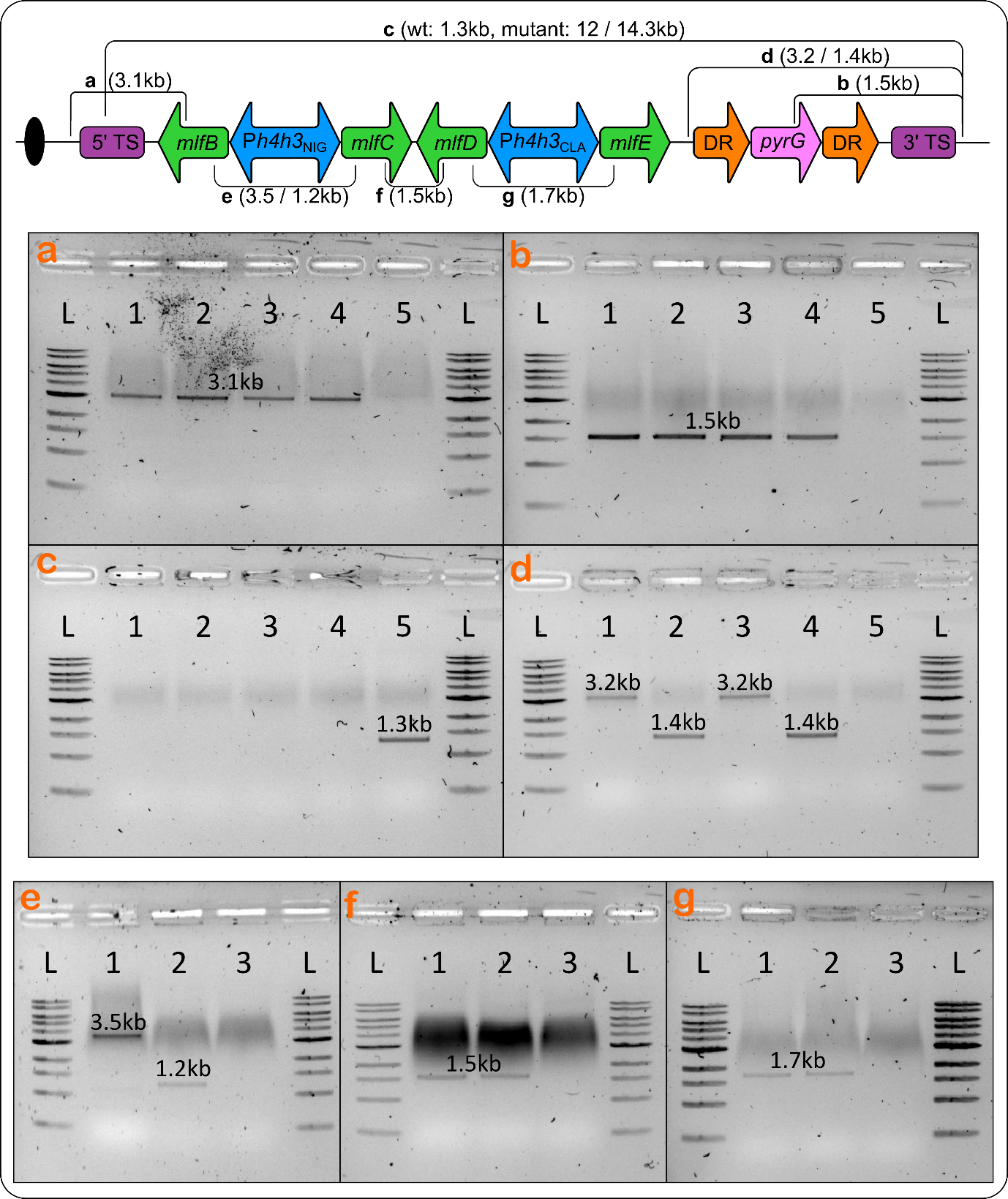


**Fig. S3** PCR validation strategy.

**Validation of strains containing the four-gene residual biosynthetic gene cluster**

Strains containing the four-gene residual biosynthetic gene cluster (rBGC and rBGC*) integrated in *A. nidulans* IS4 (top). This PCR validation strategy illustrates the DNA regions spanned by each reaction (a-g), with respect to the inserted construct (between Up- and downstream targeting sequences, TSs) and the unique sequences outside the construct, with the expected band length indicated in parenthesis. The PCR results for each reaction (a-g) are shown in the individual gel-electrophoresis images (**a**-**g**), where each lane show the PCR result for a given strain for that specific reaction. In **a**-**g**, the last of the numbered lanes displays the result obtained from the respective PCR reaction with wild-type *A. nidulans* gDNA as template, and the two outermost lanes contain 1 kb DNA ladders (L).

Reactions **a**-**c** were conducted on gDNA of strains containing the four-gene constructs rBGC [NID2412-13] and rBGC* [NID2418-19], in lanes 1-4 of **a**-**c**. Reactions **a** (P82+P66; 3.1 kb) and **b** (P78+P83; 1.5 kb) validate correct gene targeting by spanning from either *mlfB* or the *pyrG* marker, across the respective TSs, into the unique sequences of the integration site. Reaction **c** (P78+P77) examined whether strains were homokaryons by spanning across the inserted construct, as wild-type loci would yield a 1.3 kb band (obtained for wild-type gDNA, lane 5 in **c**) while mutant loci should yield a 12-14.3 kb band (dependent whether rBGC* or rBGC, respectively). As none of the strains displayed the wild-type band, the strains were concluded to be homokaryons. Reaction **d** (P78+P84) was conducted on gDNA of strains containing rBGC [NID2413+14] and rBGC* [NID2419+20], which before excision of the *pyrG* marker yields a 3.2 kb band (Lane 1+3), and a 1.4 kb band after *pyrG* excision (Lane 2+4), thereby providing genotypic validation of *pyrG* marker excision.

Reactions **e**-**g** were conducted on gDNA of strains rBGC [NID2414] and rBGC* [NID2420] after *pyrG* excision, and validated the integrity of the constructs by each spanning a certain region of the cassette. Lane 1: rBGC [NID2414], Lane 2: rBGC* [NID2420], Lane 3: wild-type *A. nidulans* gDNA. Reaction **e** (P85+P86) span the first P*h4h3* (NIG) as the primers bind in 5’ end of *mlfB* and the middle of *mlfC* (corresponding to the 5’ of *mlfC** in rBGC*), thereby yielding a 3.5 kb for construct rBGC (Lane 1) and 1.2 kb band of rBGC* (Lane 2). The second P*h4h3* (CLA) is covered by reaction **g** (P89+P90) as the primers bind in the 5’ end of *mlfD* and *mlfE*, thereby yielding a 1.7 kb band for both rBGC (Lane 1) and rBGC* (Lane 2). Reaction **f** (P87+P88) spans the 3’ ends of *mlfC*/*mlfC** and *mlfD*, yielding a 1.5 kb band for both rBGC (Lane 1) and rBGC* (Lane 2).

**Validation of *mlfA* overexpression strains and reporter strains**

The *mlfA* overexpression strains and the reporter strains were validated for correct targeted integration and homokaryon using the PCR strategies described above, specifically using reaction **b** and **c**, as detailed below (data not shown).

The construct overexpressing the malformin biosynthetic gene *mlfA* (pAC1380) was integrated in IS1, in the parental strains rBGC [NID2414], rBGC* [NID2420], and NID1. Biological triplicates of each transformation [NID2415-17, NID2421-23, and NID2424+2439-40, respectively] were validated. Targeted integration in *A. nidulans* IS1 was validated by reaction **b** (P83+P81), yielding a 2.5 kb band (data not shown). Strains were validated as homokaryons by reaction **c** (P79+P80), which should yield a 1.4 kb band for wild-type loci and 22 kb for mutant loci, of which the 1.4 kb wild-type sized band could only be obtained from wild-type gDNA.

Strains containing the P*h3h4* reporter constructs [NID2346-78] and reference promoter constructs [NID2343-45, NID2383-85, NID2389-91, NID2395-97] were validated for targeted integration in IS4 by PCR reaction **b** (P78+P83) producing a 1.5 kb band (data not shown). Strains validated as homokaryons by reaction **c** (P77+P78), which should yield a 1.3 kb band for intact wild-type loci and 7 kb for planned targeted loci, of which the 1.3 kb wild-type sized band was only obtained from wild-type gDNA.


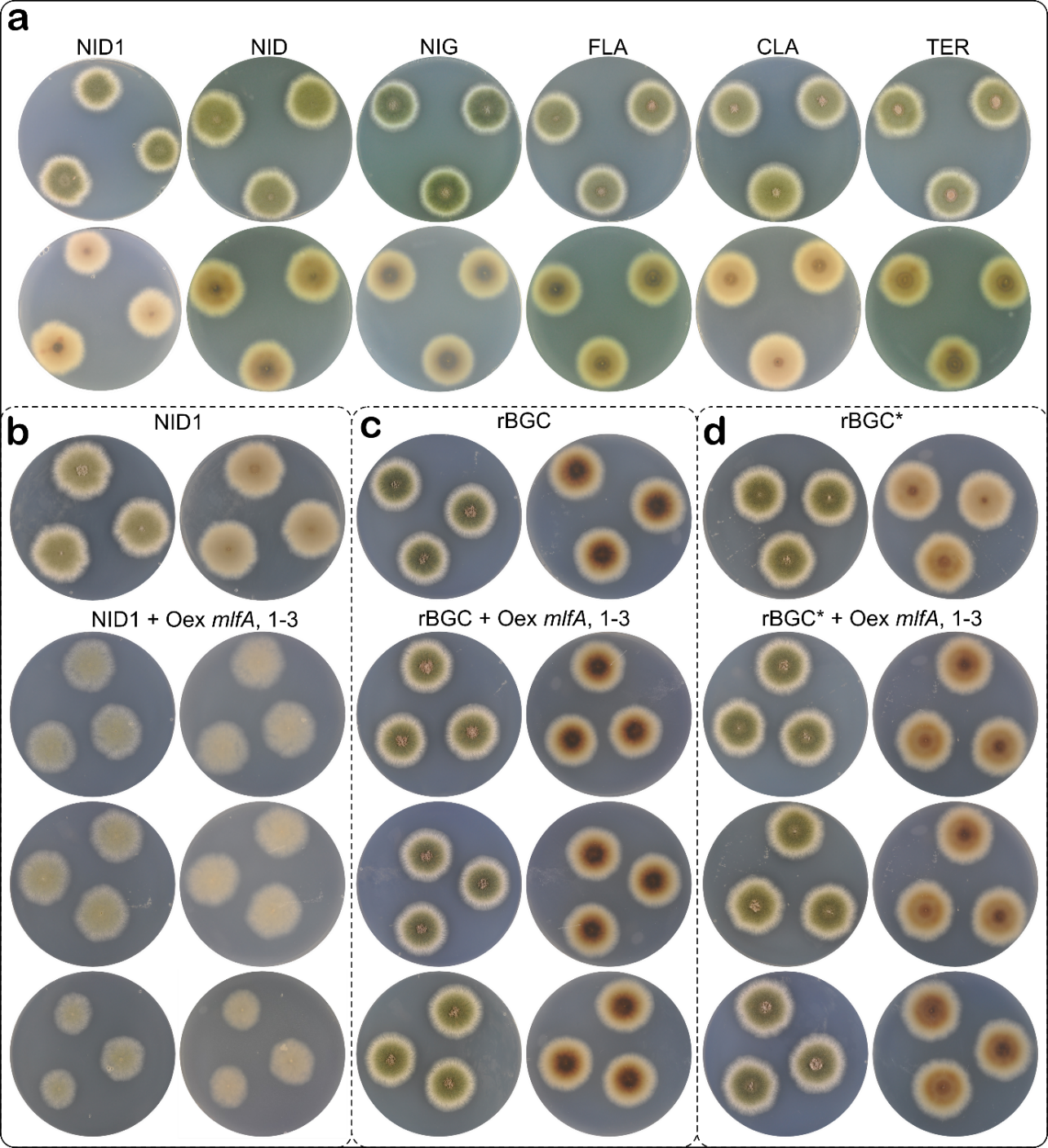


**Figure S4** Key *A. nidulans* mutant strains as three-point inoculations cultivated for 3 days at 37^o^C on minimal media with necessary supplements. (**a**) The parental strain (NID1) and representative reporter strains expressing mRFP and mCitrine from the P*h4h3* of *A. nidulans* (NID) [NID2346], *A. niger* (NIG) [NID2349], *A. flavus* (FLA) [NID2352], *A. clavatus* (CLA) [NID2355], and *A. terreus* (TER) [NID2358], with forward and reverse picture at the top and bottom, respectively. The panels **b**-**d** show the triplicate *mlfA* overexpression strains (Oex *mlfA*, 1-3) in the genetic backgrounds of the three parental strains; NID1 (**b**), rBGC [NID2414] (**c**), and rBGC* [NID2420] (**d**), with forward and reverse pictures placed to the left and right, respectively.

In panel (**a**), the P*h4h3* reporter strains displayed no morphological change compared to the parental strains (NID1). In panel (**b**), the Oex *mlfA* strains display a thinner mycelium, weaker sporulation and slightly decreased colony diameter compared to the parental strain NID1. In panels (**c**) and (**d**), the Oex *mlfA* strains display no morphological change compared to the parental strains rBGC and rBGC*, respectively. However, a more intense coloration on reverse is observed for both rBGC, rBGC*, and the related Oex *mlfA* strains when compared to the original parental strain NID1. No change in colony diameter is observed between the strains in (**b**) and (**c**) compared to NID1.

**
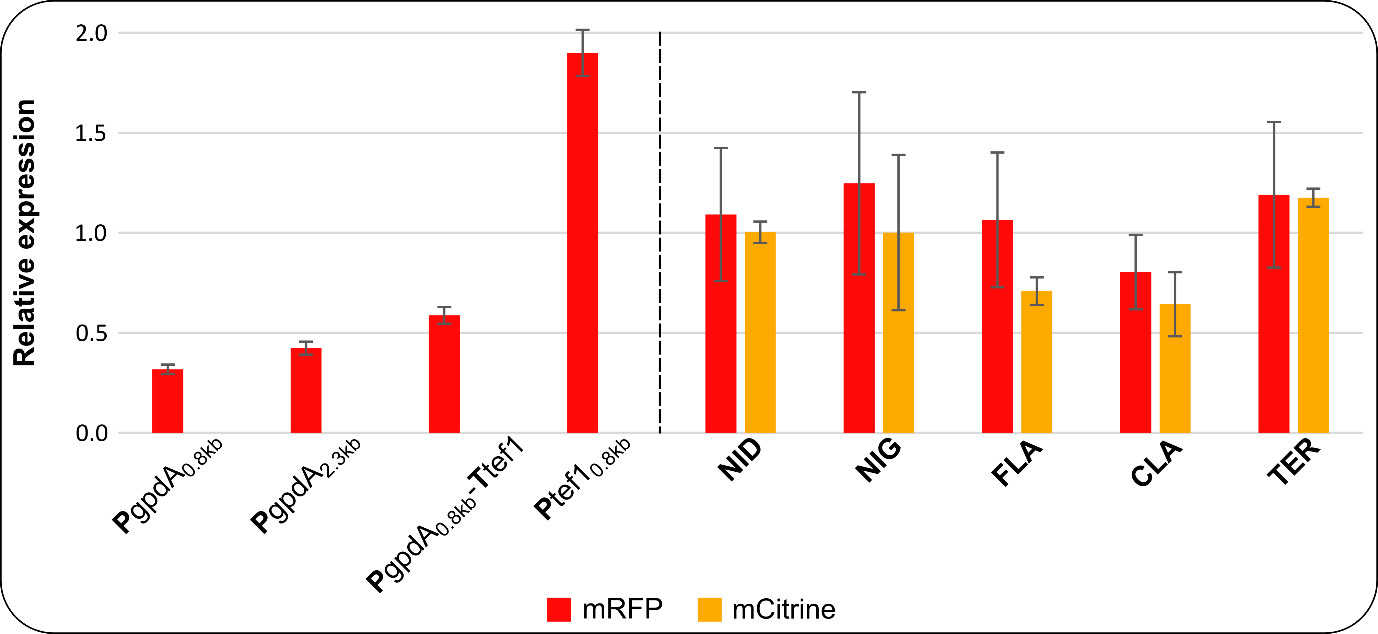
**

**Figure S5** Relative expression from promoters during solid stage cultivation. Relative expression determined by RT-qPCR of mRFP (red) expressed by the four reference constructs (left) with promoters; P*gpdA*_0.8kb_ [NID2343-45], P*gpdA*_2.3kb_ [NID2383-85], P*gpdA*_0.8kb_-T*tef1*[NID2389-91], and P*tef1*_0.8kb_ [NID2395-97]. Transcriptional termination occured by T*trpC*, with the exception of P*gpdA* _0.8kb_-T*tef1*, where T*tef1* was applied. The five P*h4h3* promoter constructs (right), expressing mRFP (red, P*h4*’) and mCitrine (yellow, P*h3*’) from the P*h4h3* of *A. nidulans* (NID) [NID2346-48], *A. niger* (NIG) [NID2349-51], *A. flavus* (FLA) [NID2352-54], *A. clavatus* (CLA) [NID2355-57], and *A. terreus* (TER) [NID2358-60]. Error bars represent standard error of the mean.


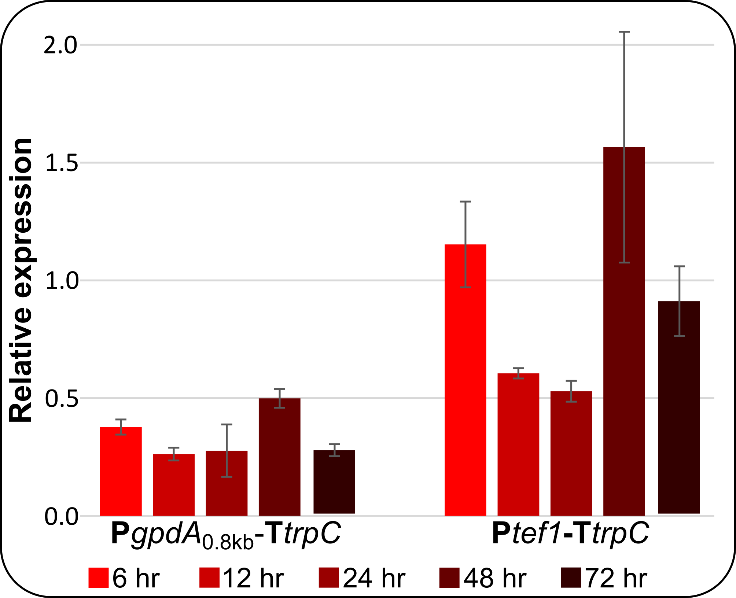


**Figure S6** Relative expression from reference promoters during submerged cultivation. RT-qPCR relative expression of mRFP from strains with reference promoters P*gpdA* [NID2343-45] and P*tef1* [NID2395-2397] during submerged cultivation at different time-points (6, 12, 24, 48, and 72 hours), before normalization to gDW/L. Error bars represent standard error of the mean.

**
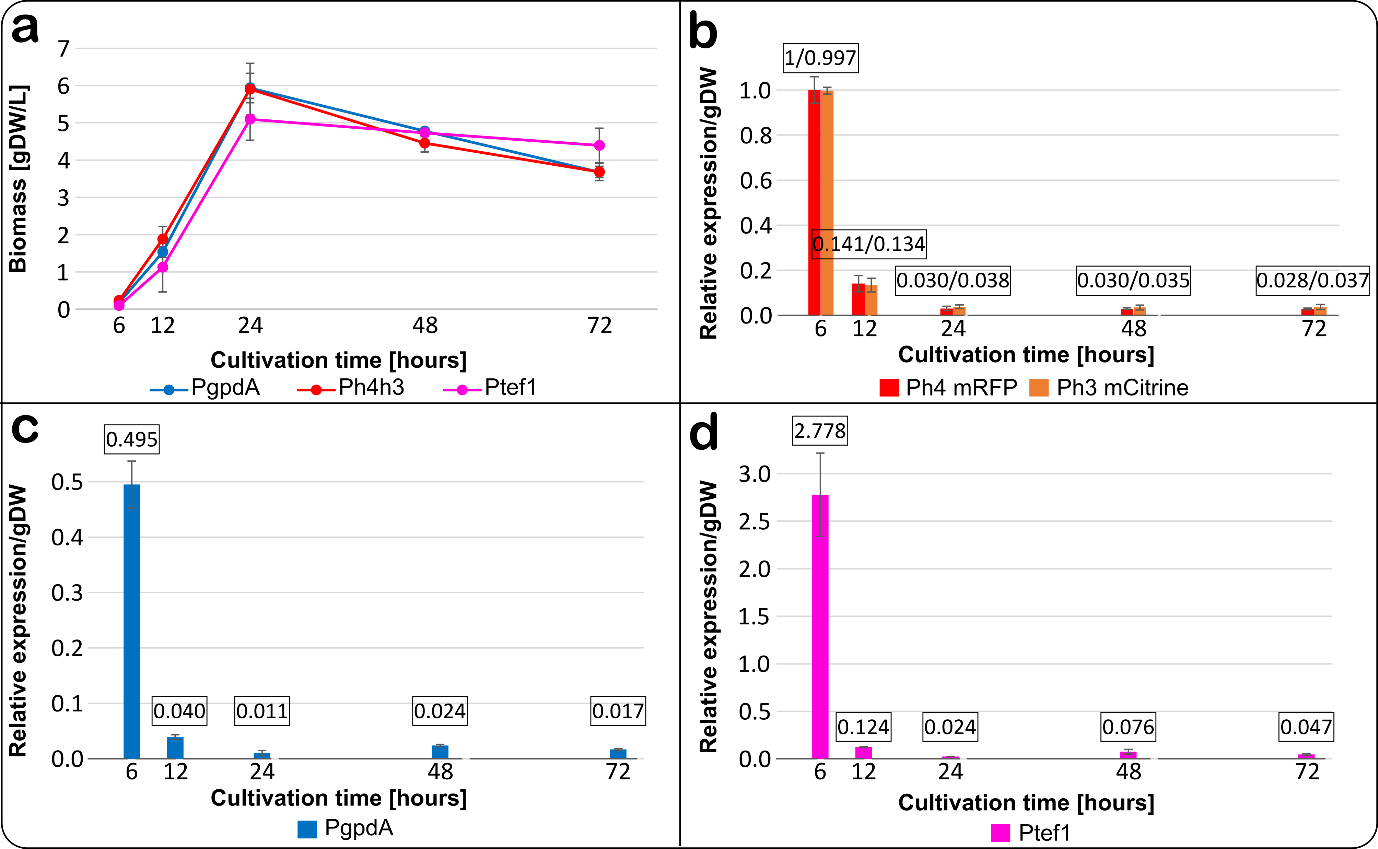
**

**Figure S7** Biomass concentration during submerged cultivation and relative expression from promoters normalized to biomass concentration. Biomass concentration (gDW/L) of reporter strains expressing mRFP and mCitrine from P*h4h3* NID [NID2376-2378], and the reference strains expressing mRFP from P*gpdA* [NID2343-45] and P*tef1* [NID2395-2397] during submerged cultivation (**a**). The qRT-PCR relative expression during submerged cultivation after normalization to gDW/L, from promoters P*h4h3* (**b**), P*gpdA* (**c**), and P*tef1* (**d**). The strains equipped with the expression constructs based on the respective three promoters were each grown in one shake-flask per time-point; 6, 12, 24, 48 and 72 hours. At each time-point, samples were collected for DW measurement (5mL, in triplicate) and RNA extraction (1mL), and the shake-flask discarded. The relative expression determined by RT-qPCR before normalization to gDW/L are shown for P*h4h3* (in main text, Fig. 4), and the reference promoters P*gpdA* and P*tef1* (in additional file 1, Fig.2). Error bars represent standard error of the mean.

**Exclusion of reporter construct integration at endogenous loci of P*h4h3*, T*trpC*, and T*tef1***

Since one of the applied P*h4h3*s and both terminators (T*trpC* and T*tef1*) are endogenous to *A. nidulans*, we ensured that the reporter constructs with the P*h4h3* of *A. nidulans* had not integrated at the loci of the P*h4h3* promoter or the T*trpC* and T*tef1* terminators. gDNA of the triplicate strains with the type *a* constructs using the P*h4h3* of *A. nidulans* (NID2346-48) were applied for diagnostic PCR. To verify the integrity of the native P*h4h3* locus in *A. nidulans*, two PCR reaction were made (P99+P100: 883bp; P101+P102: 690bp), spanning from either the P*h4’* end or the P*h3’* end of the P*h4h3* across the respective histone gene (*h4.1* or *h3*) and into the downstream region. Similarly for T*trpC* and T*tef1* (P103+P104: 2096bp; P105+P016: 2061bp), we performed PCR reactions amplifying the respective genes (*trpC* and *tef1*) and downstream of the terminator sequences. As expected, for all three strains, the four reactions showed intact loci (data not shown).
