# Supplementary material for: Bidirectional histone-gene promoters in Aspergillus: characterization and application for multi-gene expression
Source: Fungal Biol Biotechnol. 2019 Dec 9;6:24. doi: 10.1186/s40694-019-0088-3 (PMC6900853; doi:10.1186/s40694-019-0088-3)
Supplement: Supplementary file 6 — Additional file 6. Tables of Strains, Primers, Plasmids and USER fragments. Table S7. Strains used in this study. Table S8. Primers used in this study. Table S9. Plasmids used in this study. Table S10. USER fragments applied in this study. [file 40694_2019_88_MOESM6_ESM.docx]

**Additional file 6.** Tables of Strains, Primers, Plasmids and USER fragments.

**Table S7** Strains used in this study

| **Strain ID** | **Genotype** | **Source** |
| --- | --- | --- |
| **Parental strain** | | |
| NID1/IBT29539 | *argB2, pyrG89, veA1, nkuA*Δ | Nielsen et al. 2008 |
| **P*h4h3* reporter strains** | | |
| NID2346 | IS4::T*tef1-mRFP-*P*h4h3* NID*-mCitrine-*T*trpC*-DR-AFUM*pyrG*-DR | This study |
| NID2347 | IS4::T*tef1-mRFP-*P*h4h3* NID*-mCitrine-*T*trpC*-DR-AFUM*pyrG*-DR | This study |
| NID2348 | IS4::T*tef1-mRFP-*P*h4h3* NID*-mCitrine-*T*trpC*-DR-AFUM*pyrG*-DR | This study |
| NID2349 | IS4::T*tef1-mRFP-*P*h4h3* NIG*-mCitrine-*T*trpC*-DR-AFUM*pyrG*-DR | This study |
| NID2350 | IS4::T*tef1-mRFP-*P*h4h3* NIG*-mCitrine-*T*trpC*-DR-AFUM*pyrG*-DR | This study |
| NID2351 | IS4::T*tef1-mRFP-*P*h4h3* NIG*-mCitrine-*T*trpC*-DR-AFUM*pyrG*-DR | This study |
| NID2352 | IS4::T*tef1-mRFP-*P*h4h3* FLA*-mCitrine-*T*trpC*-DR-AFUM*pyrG*-DR | This study |
| NID2353 | IS4::T*tef1-mRFP-*P*h4h3* FLA*-mCitrine-*T*trpC*-DR-AFUM*pyrG*-DR | This study |
| NID2354 | IS4::T*tef1-mRFP-*P*h4h3* FLA*-mCitrine-*T*trpC*-DR-AFUM*pyrG*-DR | This study |
| NID2355 | IS4::T*tef1-mRFP-*P*h4h3* CLA*-mCitrine-*T*trpC*-DR-AFUM*pyrG*-DR | This study |
| NID2356 | IS4::T*tef1-mRFP-*P*h4h3* CLA*-mCitrine-*T*trpC*-DR-AFUM*pyrG*-DR | This study |
| NID2357 | IS4::T*tef1-mRFP-*P*h4h3* CLA*-mCitrine-*T*trpC*-DR-AFUM*pyrG*-DR | This study |
| NID2358 | IS4::T*tef1-mRFP-*P*h4h3* TER*-mCitrine-*T*trpC*-DR-AFUM*pyrG*-DR | This study |
| NID2359 | IS4::T*tef1-mRFP-*P*h4h3* TER*-mCitrine-*T*trpC*-DR-AFUM*pyrG*-DR | This study |
| NID2360 | IS4::T*tef1-mRFP-*P*h4h3* TER*-mCitrine-*T*trpC*-DR-AFUM*pyrG*-DR | This study |
| NID2361 | IS4::T*trpC-mCitrine-*P*h4h3* NID*-mRFP-*T*tef1*-DR-AFUM*pyrG*-DR | This study |
| NID2362 | IS4::T*trpC-mCitrine-*P*h4h3* NID*-mRFP-*T*tef1*-DR-AFUM*pyrG*-DR | This study |
| NID2363 | IS4::T*trpC-mCitrine-*P*h4h3* NID*-mRFP-*T*tef1*-DR-AFUM*pyrG*-DR | This study |
| NID2364 | IS4::T*trpC-mCitrine-*P*h4h3* NIG*-mRFP-*T*tef1*-DR-AFUM*pyrG*-DR | This study |
| NID2365 | IS4::T*trpC-mCitrine-*P*h4h3* NIG*-mRFP-*T*tef1*-DR-AFUM*pyrG*-DR | This study |
| NID2366 | IS4::T*trpC-mCitrine-*P*h4h3* NIG*-mRFP-*T*tef1*-DR-AFUM*pyrG*-DR | This study |
| NID2367 | IS4::T*trpC-mCitrine-*P*h4h3* FLA*-mRFP-*T*tef1*-DR-AFUM*pyrG*-DR | This study |
| NID2368 | IS4::T*trpC-mCitrine-*P*h4h3* FLA*-mRFP-*T*tef1*-DR-AFUM*pyrG*-DR | This study |
| NID2369 | IS4::T*trpC-mCitrine-*P*h4h3* FLA*-mRFP-*T*tef1*-DR-AFUM*pyrG*-DR | This study |
| NID2370 | IS4::T*trpC-mCitrine-*P*h4h3* CLA*-mRFP-*T*tef1*-DR-AFUM*pyrG*-DR | This study |
| NID2371 | IS4::T*trpC-mCitrine-*P*h4h3* CLA*-mRFP-*T*tef1*-DR-AFUM*pyrG*-DR | This study |
| NID2372 | IS4::T*trpC-mCitrine-*P*h4h3* CLA*-mRFP-*T*tef1*-DR-AFUM*pyrG*-DR | This study |
| NID2373 | IS4::T*trpC-mCitrine-*P*h4h3* TER*-mRFP-*T*tef1*-DR-AFUM*pyrG*-DR | This study |
| NID2374 | IS4::T*trpC-mCitrine-*P*h4h3* TER*-mRFP-*T*tef1*-DR-AFUM*pyrG*-DR | This study |
| NID2375 | IS4::T*trpC-mCitrine-*P*h4h3* TER*-mRFP-*T*tef1*-DR-AFUM*pyrG*-DR | This study |
| NID2376 | IS4::T*trpC-mRFP-*P*h4h3* NID*-mCitrine-*T*tef1*-DR-AFUM*pyrG*-DR | This study |
| NID2377 | IS4::T*trpC-mRFP-*P*h4h3* NID*-mCitrine-*T*tef1*-DR-AFUM*pyrG*-DR | This study |
| NID2378 | IS4::T*trpC-mRFP-*P*h4h3* NID*-mCitrine-*T*tef1*-DR-AFUM*pyrG*-DR | This study |
|  |  |  |
|  |  |  |
|  |  |  |
|  |  |  |
|  |  |  |
|  |  |  |
| ***Table S7****, continued* | | |
| **Reference promoter constructs** | | |
| NID2343 | IS4::P*gpdA*_0.8kb_*-mRFP-*T*trpC*-DR-AFUM*pyrG*-DR | This study |
| NID2344 | IS4::P*gpdA*_0.8kb_*-mRFP-*T*trpC*-DR-AFUM*pyrG*-DR | This study |
| NID2345 | IS4::P*gpdA*_0.8kb_*-mRFP-*T*trpC*-DR-AFUM*pyrG*-DR | This study |
| NID2383 | IS4::P*gpdA*_2.3kb_*-mRFP-*T*trpC*-DR-AFUM*pyrG*-DR | This study |
| NID2384 | IS4::P*gpdA*_2.3kb_*-mRFP-*T*trpC*-DR-AFUM*pyrG*-DR | This study |
| NID2385 | IS4::P*gpdA*_2.3kb_*-mRFP-*T*trpC*-DR-AFUM*pyrG*-DR | This study |
| NID2389 | IS4::P*gpdA*_0.8kb_*-mRFP-*T*tef1*-DR-AFUM*pyrG*-DR | This study |
| NID2390 | IS4::P*gpdA*_0.8kb_*-mRFP-*T*tef1*-DR-AFUM*pyrG*-DR | This study |
| NID2391 | IS4::P*gpdA*_0.8kb_*-mRFP-*T*tef1*-DR-AFUM*pyrG*-DR | This study |
| NID2395 | IS4::P*tef1-mRFP-*T*trpC*-DR-AFUM*pyrG*-DR | This study |
| NID2396 | IS4::P*tef1-mRFP-*T*trpC*-DR-AFUM*pyrG*-DR | This study |
| NID2397 | IS4::P*tef1-mRFP-*T*trpC*-DR-AFUM*pyrG*-DR | This study |
| **Malformin pathway experssion strains** | | |
| NID2412 | IS4::*mlfB-*P*h4h3* NIG*-mlfC-mlfD-*P*h4h3* CLA*-mlfE*-DR-AFUM*pyrG*-DR | This study |
| NID2413 | IS4::*mlfB-*P*h4h3* NIG*-mlfC-mlfD-*P*h4h3* CLA*-mlfE*-DR-AFUM*pyrG*-DR | This study |
| NID2414 | IS4::*mlfB-*P*h4h3* NIG*-mlfC-mlfD-*P*h4h3* CLA*-mlfE* | This study |
| NID2415 | IS4::*mlfB-*P*h4h3* NIG*-mlfC-mlfD-*P*h4h3* CLA*-mlfE*, IS1::P*gpdA*-*mlfA*-T*trpC*-DR-AFUM*pyrG*-DR | This study |
| NID2416 | IS4::*mlfB-*P*h4h3* NIG*-mlfC-mlfD-*P*h4h3* CLA*-mlfE*, IS1::P*gpdA*-*mlfA*-T*trpC*-DR-AFUM*pyrG*-DR | This study |
| NID2417 | IS4::*mlfB-*P*h4h3* NIG*-mlfC-mlfD-*P*h4h3* CLA*-mlfE*, IS1::P*gpdA*-*mlfA*-T*trpC*-DR-AFUM*pyrG*-DR | This study |
| NID2418 | IS4::*mlfB-*P*h4h3* NIG*-mlfC*-mlfD-*P*h4h3* CLA*-mlfE*-DR-AFUM*pyrG*-DR | This study |
| NID2419 | IS4::*mlfB-*P*h4h3* NIG*-mlfC*-mlfD-*P*h4h3* CLA*-mlfE*-DR-AFUM*pyrG*-DR | This study |
| NID2420 | IS4::*mlfB-*P*h4h3* NIG*-mlfC*-mlfD-*P*h4h3* CLA*-mlfE* | This study |
| NID2421 | IS4::*mlfB-*P*h4h3* NIG*-mlfC*-mlfD-*P*h4h3* CLA*-mlfE*, IS1::P*gpdA*-*mlfA*-T*trpC*-DR-AFUM*pyrG*-DR | This study |
| NID2422 | IS4::*mlfB-*P*h4h3* NIG*-mlfC*-mlfD-*P*h4h3* CLA*-mlfE*, IS1::P*gpdA*-*mlfA*-T*trpC*-DR-AFUM*pyrG*-DR | This study |
| NID2423 | IS4::*mlfB-*P*h4h3* NIG*-mlfC*-mlfD-*P*h4h3* CLA*-mlfE*, IS1::P*gpdA*-*mlfA*-T*trpC*-DR-AFUM*pyrG*-DR | This study |
| NID2424 | IS1::P*gpdA-mlfA-*T*trpC*-DR-AFUMpyrG-DR | This study |
| NID2439 | IS1::P*gpdA-mlfA-*T*trpC*-DR-AFUMpyrG-DR | This study |
| NID2440 | IS1::P*gpdA-mlfA-*T*trpC*-DR-AFUMpyrG-DR | This study |
| **Ph4h3 internal truncation** | | |
| NID2525 | IS4::T*trpC-mCitrine-*P*h4h3* NID_400bp_*-mRFP-*T*tef1*-DR-AFUM*pyrG*-DR | This study |
| NID2526 | IS4::T*trpC-mCitrine-*P*h4h3* NID_400bp_*-mRFP-*T*tef1*-DR-AFUM*pyrG*-DR | This study |
| NID2527 | IS4::T*trpC-mCitrine-*P*h4h3* NID_400bp_*-mRFP-*T*tef1*-DR-AFUM*pyrG*-DR | This study |
| NID2528 | IS4::T*trpC-mCitrine-*P*h4h3* NID_200bp_*-mRFP-*T*tef1*-DR-AFUM*pyrG*-DR | This study |
| NID2529 | IS4::T*trpC-mCitrine-*P*h4h3* NID_200bp_*-mRFP-*T*tef1*-DR-AFUM*pyrG*-DR | This study |
| NID2530 | IS4::T*trpC-mCitrine-*P*h4h3* NID_200bp_*-mRFP-*T*tef1*-DR-AFUM*pyrG*-DR | This study |

All strains were generated in the genetic background of the parental strain NID1 (IBT 29539). The excision of the AFUM*pyrG* marker from strains NID2413 and NID2419 led to strains NID2414 and NID2420, which were parental strains of NID2415-2417 and NID2421-2423, respectively.

**Table S8** Primers used in this study

| **Code** | **Name** | **Sequence (5'-3')** |
| --- | --- | --- |
| **Ph4h3 amplification** | |  |
| P1 | Promoter-histonedual-F | ATGTTGAUGTGTGAAGAGATTTAAGGT |
| P2 | Promoter-histonedual-R | ATTATTGACGAUGAGTTTTGATGGAATTAG |
| P3 | ANIG-Ph3h4-FU | ATTGTGAAGGUTGTCTAAAAAGTAAACG |
| P4 | ANIG-Ph3h4-RU | ATCTTAGUGGATTAAGTTTGATGGATTTAG |
| P5 | AFLA-Ph3h4-FU | ATATTGAUGGTTGATTAAAGAGTTAACTTG |
| P6 | AFLA-Ph3h4-RU | ATCTTTAGAGAUAAGGTTTGATGGATTTAG |
| P7 | ACLA-Ph3h4-FU | ATTTTGAUAGATGTGAATTAAAAAAGGTTTGC |
| P8 | ACLA-Ph3h4-RU | ATTTTTGAUGATTAGGTTTGATGGATTTAGTTG |
| P9 | ATER-Ph3h4-FU | ATGTTGACGGUTGTGTATTAGAAGGTTG |
| P10 | ATER-Ph3h4-RU | ATTGTAGUGGATTAGATTTGATGGATTTG |
| **Normal orientation constructs** | |  |
| P11 | Ttef-mRFP-RU-PacI Dw | GGTCTTAAUGTATTGGGATGAATTTTGTATGC |
| P12 | Ttef-mRFP-FU-AN Ph3h4 | ATCAACAUGGCCTCCTCCGAGGACGTC |
| P13 | Ttef-mRFP-FU-ANI Ph3h4 | ACCTTCACAAUGGCCTCCTCCGAGGACGTC |
| P14 | Ttef-mRFP-FU-AFLA Ph3h4 | ATCAATAUGGCCTCCTCCGAGGACGTC |
| P15 | Ttef-mRFP-FU-ACLA Ph3h4 | ATCAAAAUGGCCTCCTCCGAGGACGTC |
| P16 | Ttef-mRFP-FU-ATER Ph3h4 | ACCGTCAACAUGGCCTCCTCCGAGGACGTC |
| P17 | TtrpC-mCit-RU-PacI Up | GGGTTTAAUCGCTTACACAGTACACGAGG |
| P18 | TtrpC-mCit-FU-AN ph3h4 | ATCGTCAATAAUGGTGAGCAAGGGCGAGGAG |
| P19 | TtrpC-mCit-FU-ANI ph3h4 | ACTAAGAUGGTGAGCAAGGGCGAGGAG |
| P20 | TtrpC-mCit-FU-AFLA ph3h4 | ATCTCTAAAGAUGGTGAGCAAGGGCGAGGAG |
| P21 | TtrpC-mCit-FU-ACLA ph3h4 | ATCAAAAAUGGTGAGCAAGGGCGAGGAG |
| P22 | TtrpC-mCit-FU-ATER ph3h4 | ACTACAAUGGTGAGCAAGGGCGAGGAG |
| **Reverse orientation constructs** | |  |
| P23 | Ttef-mRFP-RU-PacI Up | GGGTTTAAUGTATTGGGATGAATTTTGTATGC |
| P24 | Ttef-mRFP-FU-AN ph3h4 | ATCGTCAATAAUGGCCTCCTCCGAGGACGTC |
| P25 | Ttef-mRFP-FU-ANI ph3h4 | ACTAAGAUGGCCTCCTCCGAGGACGTC |
| P26 | Ttef-mRFP-FU-AFLA ph3h4 | ATCTCTAAAGAUGGCCTCCTCCGAGGACGTC |
| P27 | Ttef-mRFP-FU-ACLA ph3h4 | ATCAAAAAUGGCCTCCTCCGAGGACGTC |
| P28 | Ttef-mRFP-FU-ATER ph3h4 | ACTACAAUGGCCTCCTCCGAGGACGTC |
| P29 | TtrpC-mCit-RU-PacI Dw | GGTCTTAAUCGCTTACACAGTACACGAGG |
| P30 | TtrpC-mCit-FU-AN Ph3h4 | ATCAACAUGGTGAGCAAGGGCGAGGAG |
| P31 | TtrpC-mCit-FU-ANI Ph3h4 | ACCTTCACAAUGGTGAGCAAGGGCGAGGAG |
| P32 | TtrpC-mCit-FU-AFLA Ph3h4 | ATCAATAUGGTGAGCAAGGGCGAGGAG |
| P33 | TtrpC-mCit-FU-ACLA Ph3h4 | ATCAAAAUGGTGAGCAAGGGCGAGGAG |
| P34 | TtrpC-mCit-FU-ATER Ph3h4 | ACCGTCAACAUGGTGAGCAAGGGCGAGGAG |
| **Terminator switch construct** | |  |
| P35 | Ttef-mRFP-FU-AN ph3h4 | ATCGTCAATAAUGGCCTCCTCCGAGGACGTC |
| P36 | mRFP-RU-TtrpC | AGTGGATCCUTAGGCGCCGGTGGAGTG |
| P37 | Ttef-FU-mCitrine | AGGCGGACAUTCGATTTATGC |
| P38 | Ttef-mRFP-RU-PacI Up | GGGTTTAAUGTATTGGGATGAATTTTGTATGC |
| P39 | TtrpC-mCit-FU-AN Ph3h4 | ATCAACAUGGTGAGCAAGGGCGAGGAG |
| P40 | mCitrine-RU-Ttef | ATGTCCGCCUTGTACAGCTCGTCCATG |
| P41 | TtrpC-FU-mRFP | AGGATCCACUTAACGTTAC |
| P42 | TtrpC-mCit-RU-PacI Dw | GGTCTTAAUCGCTTACACAGTACACGAGG |
|  |  |  |
|  |  |  |
|  |  |  |
|  |  |  |
|  |  |  |
|  |  |  |
| ***Table S8****, continued* | | |
| **Reference promoter constructs** | |  |
| P43 | PgpdA 0.8-FU-PacI Up | GGGTTTAAUCAGAGAGAAGGGCTGAGTAATAAG |
| P44 | PgpdA-RU-mRFP | ATGCGGUAGTGATGTCTGCTC |
| P45 | PgpdA 2.3-FU-PacI Up | GGGTTTAAUATTCCCTTGTATCTCTACACACAGG |
| P46 | TtrpC-FU-mRFP | AGGATCCACUTAACGTTAC |
| P47 | TtrpC-RU-PacI Dw | GGTCTTAAUCGCTTACACAGTACACGAGG |
| P48 | Ptef-FU-PacI Up | GGGTTTAAUCGAGACAGCAGAATCACCG |
| P49 | Ptef-RU | ATGGTGAAGGUTGTGTTATGTTTTG |
| P50 | Ttef-FU | AGCGGACAUTCGATTTATGC |
| P51 | Ttef-mRFP-RU-PacI Dw | GGTCTTAAUGTATTGGGATGAATTTTGTATGC |
| P52 | mRFP-FU-PgpdA | ACCGCAUGGCCTCCTCCGAGGACGTC |
| P53 | mRFP-FU-Ptef | ACCTTCACCAUGGCCTCCTCCGAGGACGTC |
| P54 | mRFP-RU-TtrpC | AGTGGATCCUTAGGCGCCGGTGGAGTG |
| P55 | mRFP-RU-Ttef | ATGTCCGCUTAGGCGCCGGTGGAGTG |
| ***mlfA* amplification & pathway construction** | | |
| P56 | ABRA34020-F1-FU-PacI 2 | GGGTTTAAUATGAGTCGCTTTTCCTGC |
| P57 | ABRA34020-F1-RU 2 | AGCCCCTGUCCGTGTGTT |
| P58 | ABRA34020-F2-FU | ACAGGGGCUGTTCAACACG |
| P59 | ABRA34020-F2-RU | AGCGAGAUGCGGACAGG |
| P60 | ABRA34020-F3-FU 2 | ATCTCGCUCGGTGACCAAG |
| P61 | ABRA34020-F3-RU 2 | ACTCCGUCAACTGGAACATCTC |
| P62 | ABRA34020-F4-FU 2 | ACGGAGUGGAGATGATGGTC |
| P63 | ABRA34020-F4-RU-PacI | GGTCTTAAUTCAAACACAGACACCCCGAG |
| P64 | Abra_134974 F-Ph3h4 Nig H3 | ACCTTCACAAUGCCGCGAGGTGAACCC |
| P65 | ABRA_199881-F-pH3H4 Cla | ATCAAAAAUGTCCGTCTCTGCAGCAGAC |
| P66 | ABRA_186232-F-pH3H4 Nig | ACTAAGAUGGTCCGCATATGGCCC |
| P67 | ABRA_186232-R-PacIUp | GGGTTTAAUGTGAGAGAGGCGATGGATGAG |
| P68 | Abra_161173 F-Ph3h4 Cla H4 | ATCAAAAUGACCATCACAGCCGACG |
| P69 | Abra_161173 R-PacIDw | GGTCTTAAUCCAATCTTCAGAGTCAAATACGATG |
| P70 | Abra_334277 F-Ph3h4 Nig H3 | ACCTTCACAAUGTCTCTATCCAGCGCTTCTG |
| **RT-qPCR** | |  |
| P71 | mCit-Int-Fw | GATGTGCTTCGCCCGCTAC |
| P72 | mCit-Int-Rev | GATGCCGTTCTTCTGCTTGTC |
| P73 | mRFP-Int-Fw | GATGAGGCTGAAGCTGAAGGAC |
| P74 | mRFP-Int-Rev | CTCGTTGTGGGAGGTGATGTC |
| P75 | AN6542-Int-Fw | GTGATTTCCTTCTGCATACGGTC |
| P76 | AN6542-Int-Rev | CCACGTCACCACTTTCAACTCTATC |
| **Strain verification** | |  |
| P77 | AN-IS4-ExtGap-F | CCTGTGTTTTGCTCTAGTGATGATG |
| P78 | AN-IS4-ExtGap-R | GAGTTCGTTTCTTTCTCCGTCG |
| P79 | AN-IS1-ExtGap-F | GTTTGATGATTGCTGGTCTGTGT |
| P80 | AN-IS1-ExtGap-R | GGTGTTGGGGGAGTGTCTGTAG |
| P81 | AN-IS1-Dw-chk-R2 | GAGGTGGCGGCTTCGGAG |
| P82 | AN-IS4-Chk-Up-Fmlu | CTCGTCGTCTGAAGGGTTCTATC |
| P83 | AFUMpyrG-F-DwChk | GAGTGTTGTGGAGGAAGGCTG |
| P84 | AFUMpyrG_PopChk-Fw1 | CAGCTCTGGATAACCGTATTACC |
| P85 | ABRA186232_Int_Rev | GGTTGGTTTGGGTGTTTGG |
| P86 | ABRA334277_Int_Rev | CGTTGTTTGGTGGTAGGTCTTAC |
| P87 | ABRA134974_Int_Fw | CCTCATTTCCTCGCCACAG |
| P88 | ABRA199881_Int_Fw | CGTCCTTATCTTCTTCCCTCGT |
| P89 | ABRA199881_Int_Rev | CAGGGGAAGATTGATGTAGAAGC |
| P90 | ABRA116173_Int_Rev | CGAGTGTAGTTTGCGGGATAGC |
| ***Table S8****, continued* | | |
| **Ph4h3 NID internal truncations** | |  |
| P91 | Ph3(100bp)ANID-F | ATCGTCGUATCCTTTCCGACATCCTATATTCG |
| P92 | TtrpC-mCit-RU-PacI Up | GGGTTTAAUCGCTTACACAGTACACGAGG |
| P93 | Ph4(100bp)ANID-F | ACGACGAUGCCCATCCGGTCTTCCATT |
| P94 | Ttef-mRFP-RU-PacI Dw | GGTCTTAAUGTATTGGGATGAATTTTGTATGC |
| P95 | Ph3(200bp)ANID-F | ATCGTCGUAAGACCACCCGGCTTTGC |
| P96 | TtrpC-mCit-RU-PacI Up | GGGTTTAAUCGCTTACACAGTACACGAGG |
| P97 | Ph4(200bp)ANID-F | ACGACGAUTGCCCCGCCCTCTGATTG |
| P98 | Ttef-mRFP-RU-PacI Dw | GGTCTTAAUGTATTGGGATGAATTTTGTATGC |
| **Native loci validation** | | |
| P99 | An_H4_Up_Chk-F | GAGTGATTCCAGCTCTCTCCC |
| P100 | An_H4_Up_Chk-R | CAGTCATACCGTGAACCAACC |
| P101 | An_H3_Up_Chk-F | CCATGAGAATCCGTCCAGC |
| P102 | An_H3_Up_Chk-R | ACAAATACCATCCCCCACC |
| P103 | An_TtrpC_Up_Chk-F | ACAAGAAGGATGGTGTGAAAGC |
| P104 | An_TtrpC_Up_Chk-R | GACAAGAAGGATAAAGACGCAGAT |
| P105 | An_Ttef1_Up_Chk-F | TACAAGGGTTGGGAGAAGGAG |
| P106 | An_Ttef1_Up_Chk-R | CAGGAGGTGAGATGAATGAGGA |

**Table S9** Plasmids used in this study

| **Plasmid ID** | **Application** | **Content** | **Fragments** | **Final Strains** |
| --- | --- | --- | --- | --- |
| pAC223 | Integration vector, AN IS4 | Up_IS4_-Down_IS4_ | - | - |
| pAC1190 | Integration vector, AN IS1 | Up_IS4_-P*gpdA*-T*trpC*-Down_IS4_ | - | - |
| pAC840 | PCR template | Up_ABRA IS1_-P*gpdA*-*mCitrine*-T*trpC-*Down_ABRA IS1_ | - | - |
| pAC1033 | PCR template | P*tef1*-*mRFP*-T*tef1* | - | - |
| pAC1179 | Reporter | Up_IS4_-T*tef1*-*mRFP*-P*h4h3* NID-*mCitrine*-T*trpC*-Down_IS4_ | F1+F6+F16 | NID2346-48 |
| pAC1180 | Reporter | Up_IS4_-T*tef1*-*mRFP*-P*h4h3* NIG-*mCitrine*-T*trpC*-Down_IS4_ | F2+F7+F17 | NID2349-51 |
| pAC1181 | Reporter | Up_IS4_-T*tef1*-*mRFP*-P*h4h3* FLA-*mCitrine*-T*trpC*-Down_IS4_ | F3+F8+F18 | NID2352-54 |
| pAC1182 | Reporter | Up_IS4_-T*tef1*-*mRFP*-P*h4h3* CLA-*mCitrine*-T*trpC*-Down_IS4_ | F4+F9+F19 | NID2355-57 |
| pAC1183 | Reporter | Up_IS4_-T*tef1*-*mRFP*-P*h4h3* TER-*mCitrine*-T*trpC*-Down_IS4_ | F5+F10+F20 | NID2358-60 |
| pAC1184 | Reporter | Up_IS4_-T*trpC*-*mCitrine*-P*h4h3* NID-*mRFP*-T*tef1*-Down_IS4_ | F1+F11+F21 | NID2361-63 |
| pAC1185 | Reporter | Up_IS4_-T*trpC*-*mCitrine*-P*h4h3* NIG-*mRFP*-T*tef1*-Down_IS4_ | F2+F12+F22 | NID2364-66 |
| pAC1186 | Reporter | Up_IS4_-T*trpC*-*mCitrine*-P*h4h3* FLA-*mRFP*-T*tef1*-Down_IS4_ | F3+F13+F23 | NID2367-69 |
| pAC1187 | Reporter | Up_IS4_-T*trpC*-*mCitrine*-P*h4h3* CLA-*mRFP*-T*tef1*-Down_IS4_ | F4+F14+24 | NID2370-72 |
| pAC1188 | Reporter | Up_IS4_-T*trpC*-*mCitrine*-P*h4h3* TER-*mRFP*-T*tef1*-Down_IS4_ | F5+F15+F25 | NID2373-75 |
| pAC1189 | Reporter | Up_IS4_-T*trpC*-*mRFP*-P*h4h3* NID-*mCitrine*-T*tef1*-Down_IS4_ | F1+26-F29 | NID2376-78 |
| pAC1416 | Reference | Up_IS4_-P*gpdA*_2.3kb_-*mRFP*-T*trpC*-Down_IS4_ | F31+F35+F32 | NID2383-85 |
| pAC1420 | Reference | Up_IS4_-P*gpdA*_0.8kb_-*mRFP*-T*tef1*-Down_IS4_ | F30+F37+F34 | NID2389-91 |
| pAC1424 | Reference | Up_IS4_-P*tef1*-*mRFP*-T*trpC*-Down_IS4_ | F33+F36+F32 | NID2395-97 |
| pAC1426 | Reference | Up_IS4_-P*gpdA*_0.8kb_-*mRFP*-T*trpC*-Down_IS4_ | F30+F35+F32 | NID2343-45 |
| pAC1380 | Oex *mlfA*, AN IS1 | Up_IS1_-P*gpdA*-*mlfA*-T*trpC*-Down_IS1_ | F38-F41 | NID2424+2439-40 |
| pAC1595 | Residual biosynthetic gene cluster, rBCG | Up_IS4_-*mlfB*-P*h4h3* NIG-*mlfC*-*mlfD*-P*h4h3* CLA-*mlfE*-Down_IS4_ | F42-F44 | NID2412-17 |
| pAC1596 | Residual biosynthetic gene cluster, rBCG* | Up_IS4_-*mlfB*-P*h4h3* NIG-*mlfC**-*mlfD*-P*h4h3* CLA-*mlfE*-Down_IS4_ | F42+F44+F45 | NID1418-23 |
| pAC1688 | P*h4h3* NID, 400 bp | Up_IS4_-T*tef1*-*mRFP*-P*h4h3* NID_400bp_-*mCitrine*-T*trpC*-Down_IS4_ | F46+F47 | NID2525-27 |
| pAC1689 | P*h4h3* NID*,* 200 bp | Up_IS4_-T*tef1*-*mRFP*-P*h4h3* NID_200bp_-*mCitrine*-T*trpC*-Down_IS4_ | F48+F49 | NID2528-30 |

All plasmids contain an *E. coli* origin of replication (*oriC*), an ampicillin resistance gene (*AmpR*), and a *pyrG* marker from *A. fumigatus* (AFUM*pyrG*) with its native promoter and terminator sequences. The marker is flanked by 282bp direct repeat (DR) sequences for marker excision (DR-AFUM*pyrG*-DR), and is positioned upstream of the downstream targeting sequence (Down). Integration vectors contain a PacI/Nt.BbvCI restriction cassette for fragment insertion.

Plasmids pAC1179-83 and pAC1184-88 correspond to P*h4h3* reporter constructs in orientation (a) and (b) of Fig. 1, respectively. The applied genes from *A. brasiliensis* CBS 101740 encompass *mlfA*: Aspbr1_34020, *mlfB*: Aspbr1_186232, *mlfC*: Aspbr1_134974, *mlfD*: Aspbr1_199881, *mlfE*: Aspbr1_161173, *mlfC**: Aspbr1_334277.

For plasmid construction by USER cloning, the codes of applied fragments are listed corresponding to the generation of USER fragments, as listed in Table S4. The recipient vector for plasmid construction was pAC223, and the parental strain applied for protoplast transformation was NID1. With exception of plasmid pAC1380, where the recipient vector was pAC1190 and parental strains were NID1, NID2414, and NID2420. The final strains generated from transformation with each plasmid is provided.

**Table S10** USER fragments applied in this study

| **Fragment name** | **Notes** | **Template** | **Primer pair** | **Size (bp)** | **Fragment codes** |
| --- | --- | --- | --- | --- | --- |
| P*h4h3* NID | - | *A. nidulans* gDNA | P1+P2 | 799 | F1 |
| P*h4h3* NIG | - | *A. niger* gDNA | P3+P4 | 828 | F2 |
| P*h4h3* FLA | - | *A. flavus* gDNA | P5+P6 | 821 | F3 |
| P*h4h3* CLA | - | *A. clavatus* gDNA | P7+P8 | 853 | F4 |
| P*h4h3* TER | - | *A. terreus* gDNA | P9+P10 | 822 | F5 |
| mRFP-T*tef1* | NID (a) | pAC1033 | P11+P12 | 1167 | F6 |
| mRFP-T*tef1* | NIG (a) | pAC1033 | P11+P13 | 1167 | F7 |
| mRFP-T*tef1* | FLA (a) | pAC1033 | P11+P14 | 1167 | F8 |
| mRFP-T*tef1* | CLA (a) | pAC1033 | P11+P15 | 1167 | F9 |
| mRFP-T*tef1* | TER (a) | pAC1033 | P11+P16 | 1167 | F10 |
| mRFP-T*tef1* | NID (b) | pAC1033 | P23+P24 | 1167 | F11 |
| mRFP-T*tef1* | NIG (b) | pAC1033 | P23+P25 | 1167 | F12 |
| mRFP-T*tef1* | FLA (b) | pAC1033 | P23+P26 | 1167 | F13 |
| mRFP-T*tef1* | CLA (b) | pAC1033 | P23+P27 | 1167 | F14 |
| mRFP-T*tef1* | TER (b) | pAC1033 | P23+P28 | 1167 | F15 |
| mCitrine-T*trpC* | NID (a) | pAC840 | P17+P18 | 1465 | F16 |
| mCitrine-T*trpC* | NIG (a) | pAC840 | P17+P19 | 1465 | F17 |
| mCitrine-T*trpC* | FLA (a) | pAC840 | P17+P20 | 1465 | F18 |
| mCitrine-T*trpC* | CLA (a) | pAC840 | P17+P21 | 1465 | F19 |
| mCitrine-T*trpC* | TER (a) | pAC840 | P17+P22 | 1465 | F20 |
| mCitrine-T*trpC* | NID (b) | pAC840 | P29+P30 | 1465 | F21 |
| mCitrine-T*trpC* | NIG (b) | pAC840 | P29+P31 | 1465 | F22 |
| mCitrine-T*trpC* | FLA (b) | pAC840 | P29+P32 | 1465 | F23 |
| mCitrine-T*trpC* | CLA (b) | pAC840 | P29+P33 | 1465 | F24 |
| mCitrine-T*trpC* | TER (b) | pAC840 | P29+P34 | 1465 | F25 |
| mRFP | - | pAC1033 | P35+P36 | 678 | F26 |
| T*tef1* | - | pAC1033 | P37+P38 | 489 | F27 |
| mCitrine | - | pAC840 | P39+P40 | 717 | F28 |
| T*trpC* | - | pAC840 | P41+P42 | 748 | F29 |
| P*gpdA*, 0.8kb | - | pAC840 | P43+P44 | 836 | F30 |
| P*gpdA*, 2.3kb | - | pAC840 | P45+P44 | 2304 | F31 |
| T*trpC* | - | pAC840 | P46+P47 | 748 | F32 |
| P*tef1* | - | pAC1033 | P48+P49 | 886 | F33 |
| T*tef1* | - | pAC1033 | P50+P51 | 490 | F34 |
| mRFP | PgpdA-*-TtrpC | pAC1033 | P52+P54 | 678 | F35 |
| mRFP | Ptef1-*-TtrpC | pAC1033 | P53+P54 | 678 | F36 |
| mRFP | PgpdA-*-Ttef1 | pAC1033 | P52+P55 | 678 | F37 |
| mlfA (1) | Oex *mlfA* | *A. brasiliensis* gDNA | P56+P57 | 3687 | F38 |
| mlfA (2) | Oex *mlfA* | *A. brasiliensis* gDNA | P58+P59 | 3449 | F39 |
| mlfA (3) | Oex *mlfA* | *A. brasiliensis* gDNA | P60+P61 | 4604 | F40 |
| mlfA (4) | Oex *mlfA* | *A. brasiliensis* gDNA | P62+P63 | 3752 | F41 |
| mlfB | rBGC/rBGC* | *A. brasiliensis* gDNA | P66+P67 | 1876 | F42 |
| mlfC-mlfD | rBGC | *A. brasiliensis* gDNA | P64+P65 | 5879 | F43 |
| mlfE | rBGC/rBGC* | *A. brasiliensis* gDNA | P68+P69 | 1385 | F44 |
| mlfC*-mlfD | rBGC* | *A. brasiliensis* gDNA | P70+P65 | 3602 | F45 |
| P(h3' 100bp)-mCit-T*trpC* | 200bp P*h4h3* | pAC1179 | P91+P92 | 1550 | F46 |
| P(h4' 100bp)-mRFP-T*tef1* | 200bp P*h4h3* | pAC1179 | P93+P94 | 1280 | F47 |
| P(h3' 200bp)-mCit-T*trpC* | 400bp P*h4h3* | pAC1179 | P95+P96 | 1650 | F48 |
| P(h4' 200bp)-mRFP-T*tef1* | 400bp P*h4h3* | pAC1179 | P97+P98 | 1380 | F49 |

The fragments "mRFP-Ttef1" and "mCitrine-TtrpC" (F6-F25) were amplified to match a specific P*h4h3* (F1-F5; P*h4h3* NID, NIG, FLA, CLA, TER) at a specific promoter end (*h4*’ or *h3*’), in relation to the construct orientation (a or b), as illustrated in Fig. 2 in main text. Fragments F26-F29 were applied to generate pAC1189 and strains NID2376-78 containing the “P*h4h3* NID type a” construct with switched terminators.

The fragments "mRFP" (F35-F37) were amplified to match between (-*-) the designated promoter and terminator sequences (F30-F34) for generation of reference constructs expressing mRFP.

Fragments F46-F49 were amplified from pAC1179 containing the “P*h4h3* NID type a” construct, each reaction using one primer binding internally in the P*h4h3* and one primer binding in the 3’ end of a terminator, thereby allowing for internal truncation of the promoter.
